# The lincRNA *MIRAT* binds to IQGAP1 and modulates the MAPK pathway in NRAS mutant melanoma

**DOI:** 10.1038/s41598-018-27643-3

**Published:** 2018-07-19

**Authors:** Martina Sanlorenzo, Igor Vujic, Rosaura Esteve-Puig, Kevin Lai, Marin Vujic, Kevin Lin, Christian Posch, Michelle Dimon, Adrian Moy, Mitchell Zekhtser, Katia Johnston, Deborah Gho, Wilson Ho, Abhinay Gajjala, Juan Oses Prieto, Alma Burlingame, Adil Daud, Klemens Rappersberger, Susana Ortiz-Urda

**Affiliations:** 1 University of California San Francisco, Department of Dermatology, Mt. Zion Cancer Research Center, 2340 Sutter Street, N461, 94115 San Francisco, USA; 2 Department of Oncology, University of Turin, Torino, 10124 Italy; 3 Institute of Cancer Research, Department of Medicine I, Comprehensive Cancer Center, Medical University of Vienna, Vienna, 1090 Austria; 4 The Rudolfstiftung Hospital, Department of Dermatology, Vienna, 1030 Austria; 5 School of Medicine, Sigmund Freud University Vienna, Vienna, 1020 Austria; 6 Department of Pharmaceutical Chemistry, School of Pharmacy, University of California San Francisco - San Francisco, 94115 California, USA; 7 University of California-San Francisco, Mt Zion Cancer Research Center San Francisco, 94115 California, USA

## Abstract

Despite major advances in targeted melanoma therapies, drug resistance limits their efficacy. Long noncoding RNAs (lncRNAs) are transcriptome elements that do not encode proteins but are important regulatory molecules. LncRNAs have been implicated in cancer development and response to different therapeutics and are thus potential treatment targets; however, the majority of their functions and molecular interactions remain unexplored. In this study, we identify a novel cytoplasmic intergenic lincRNA (*MIRAT*), which is upregulated following prolonged MAPK inhibition in *NRAS* mutant melanoma and modulates MAPK signaling by binding to the MEK scaffold protein IQGAP1. Collectively, our results present *MIRAT’s* direct modulatory effect on the MAPK pathway and highlight the relevance of cytoplasmic lncRNAs as potential targets in drug resistant cancer.

## Introduction

Cutaneous melanoma is a highly aggressive cancer. In 2016, about 76380 individuals were diagnosed and 10130 patients succumbed to metastatic disease in the United States^1^. Mutations in the v-Raf murine sarcoma viral oncogene homolog B1 (BRAF) and neuroblastoma rat sarcoma viral oncogene homolog (NRAS) are found in 70–80% of all melanomas. These mutations cause anchorage independent activation of BRAF and NRAS proteins which lead to hyperactive signaling through their downstream cascades, tumor growth and uncontrolled proliferation^2^. Recently, small molecule inhibitors targeting mutant BRAF and/or its immediate downstream signaling member MEK marked a milestone in the treatment of metastatic melanoma. Systemic therapy with BRAF-inhibitors such as vemurafenib and dabrafenib as well as MEK inhibitors such as cobimetinib and trametinib have resulted in prolonged progression-free and overall survival^3–8^. Yet, most tumors become resistant to such targeted therapies, allowing disease progression. Several mechanisms of resistance to targeted inhibition have been described including: amplifications of receptor tyrosine kinases, RTKs (EGFR, ERBB3, IGFR1 and PDGFRb), secondary mutations in NRAS^9^, amplification of BRAFV600E or the expression of its alternative splicing variant^10^, upregulation of COT (MAP3K8)^11^, mutations in MEK, and downregulation of SOX10 and MITF^12^ among others. The role of long noncoding RNAs in such resistance, however, is still largely unknown^10,13–17^.

Long noncoding RNAs (lncRNAs) were identified as important parts of cell’s transcriptome and are thought to be regulatory elements of cancer initiation and progression. They are defined as RNA molecules longer then 200 base pairs which are transcribed but not translated to proteins^18,19^. LncRNAs are highly tissue (and tumor) specific and thus hold the potential for serving as biomarkers, prognostic indicators, and therapeutic targets^20–25^. The role of long noncoding RNAs in resistance of melanoma to targeted inhibition is poorly understood^10,13–17^.

In this study, we performed *de novo* transcriptome analyses (RNA-Seq) to identify lncRNAs differentially expressed in melanoma cells with acquired resistance to small molecule inhibitors of the MAPK cascade. By comparing treatment naïve cells to their drug resistant counterparts, we identified *MIRAT* (**M**APK **I**nhibitor **R**esistance **A**ssociated **T**ranscript), an lncRNA located on chromosome 8, significantly overexpressed in resistant cells. Analyses in *NRAS* mutant melanoma cells revealed *MIRAT’s* direct involvement in drug resistance to MAPK pathway inhibitors. Our results offer first evidence of a cytoplasmic lncRNA contributing to targeted inhibitor resistance in melanoma with the potential to open up new therapeutic avenues to further improve treatment.

## Results

### Acquired drug resistance to MAPK inhibitors in melanoma leads to the expression of the lincRNA MIRAT (MAPK Inhibitor Resistance Associated Transcript)

To assess whether acquired drug resistance changes the melanoma transcriptome, we first established cell lines with acquired resistance to small molecule inhibitors of BRAF and MEK. We treated 5 melanoma cell lines (2 NRAS^Q61^ mutant: DO4, MM415; 3 patients derived BRAF^V600E^ mutant: AV1, AV4, AV5) with increasing dosages of the MEK inhibitor trametinib and the BRAF inhibitor PLX4720. After six months of continuous drug incubation, we compared the drug sensitivities of treated clones with non-treated parental cells. Defined as a significant change in GI50 values (concentrations of drugs resulting in 50% growth inhibition relative to controls), 4 cell lines became resistant to the treatment (suffix ***RM***; **R**esistant to **M**APK inhibitor), while one cell line (AV5) remained sensitive to the treatment (Suffix ***ChrExp***; **Chr**onically **Exp**osed (Supplementary Fig. 1; for definition of resistant cell lines see material and methods). Resistant cells MM415RM, DO4RM, AV1RM and AV4RM, but not the still drug sensitive AV5ChrExp, retained high signaling through the MAPK pathway despite increasing drug concentrations, as evidenced by high phospho-ERK levels (Supplementary Fig. 1).

**Figure 1:**
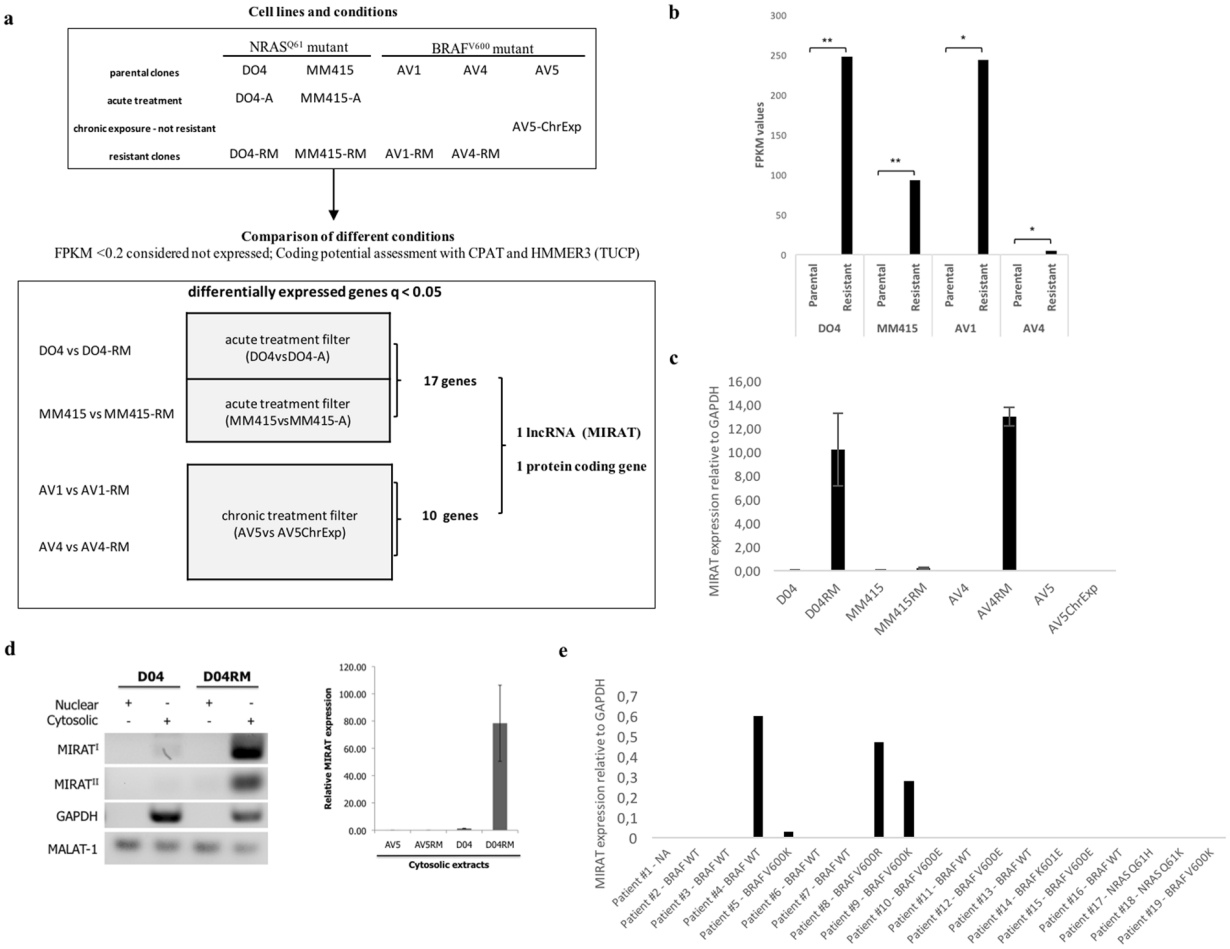
MIRAT is expressed in the cytoplasm of melanoma cells resistant to MAPK inhibitors. (**a**) Bioinformatic pipeline of RNAseq data: the comparison of resistant clones with their parental counterparts applying acute and chronic treatment filters led to the identification of one coding gene (SPOCK1) and one lncRNA (MIRAT) differently upregulated in resistant clones. Expression of MIRAT in melanoma cell lines and tissue samples was determined by PCR and RT-qPCR analyses. (**b**) Bar graph showing FPKM values of MIRAT in parental and corresponding resistant cell lines. (**c**) Bar graph displaying MIRAT expression relative to GAPDH determined by RT-qPCR analyses and real time SYBR Green amplification. (**d**) PCR and RT-qPCR analyses of MIRAT were performed in subcellular cytosolic RNA extracts of D04 and D04RM cells. The expression of MIRAT is displayed as relative values to GAPDH. (**e**) Bar graph representing MIRAT expression relative to GAPDH in 19 melanoma patients.

Next, we performed paired-end RNA sequencing from the above-mentioned cell lines and compared the transcriptomes of resistant cell lines to their parental counterparts. The initial bioinformatics analysis provided a list of differentially expressed genes (Supplementary Material and Methods). These results were further filtered with differentially expressed genes found in (i) acute (6 hours) trametinib treated cells (suffix ***A***; **A**cutely treated) and (ii) chronically exposed cells (suffix ***ChrExp***), to account for transcriptome changes after short-term and long-term drug exposures not leading to resistance (Supplementary Tables 1,2,3,4). Hierarchical clustering identified relationships among different conditions, and we found that resistant cell lines clustered together (Supplementary Fig. 2a; the heat-map illustrates the expression of differentially expressed genes). Surprisingly, after applying the different filters, only one lncRNA was differently expressed in all resistant clones (Fig. 1a,b). This lncRNA was intergenic (lincRNA) and mapped to the human chromosome 8q.24.12. We termed this transcript *MIRAT:* **M**APK **I**nhibitor **R**esistance **A**ssociated **T**ranscript (Supplementary Fig. 2b).

Probing for other, already known and probable mechanism of resistance to the MEK inhibitor trametinib we did not detect any additional NRAS mutations, SOX10 and MITF were not downregulated, and COT, BRAF and RTKs were not significantly upregulated in both NRAS mutant resistant cell lines. We did not find mutations in the coding regions of MEK1/2 and MEK1/2 RNA expression values and protein levels were comparable between parental and resistant cells (Supplementary Fig. 3)^26^. Due to low proliferation rates of patient derived avatar cell lines we performed all further experiments only in *NRAS* mutant DO4 and MM415 cell lines.

### MIRAT is enriched in the cytoplasmatic compartment of MEK inhibitor resistant NRAS mutant melanoma cell lines and is found in patient samples

We focused our efforts on the further characterization of the newly identified transcript. We confirmed *MIRAT’s* presence in both NRAS and BRAF mutant resistant cells by reverse transcription quantitative PCR (RT-qPCR) (Fig. 1c), and verified its exact sequence by Rapid Amplification of cDNA ends analysis (RACE). *MIRAT* has a 5′ cap and 3′ polyadenylated tail, indicating a transcription by RNA polymerase II. The predicted secondary structure of MIRAT is depictured in Supplementary Fig. 4.^27^ Subcellular RNA isolation showed that *MIRAT* is primarily localized in the cytoplasmic cell compartment, indicating transcript stability (Fig. 1d).

We found *MIRAT* exons expressed in 42 out of 226 (18,6%) TCGA melanoma patient samples included in the Atlas of ncRNA in Cancer (TANRIC)^28^ (Supplementary Table 5) and in 4 out of 19 (21,1%) melanoma biopsies from patients treated at our institution (21,1%) (Fig. 1e, Supplementary Table 6).

### MIRAT is upregulated in a time- and dose-dependent manner in response to MAPK inhibition

**Figure 2:**
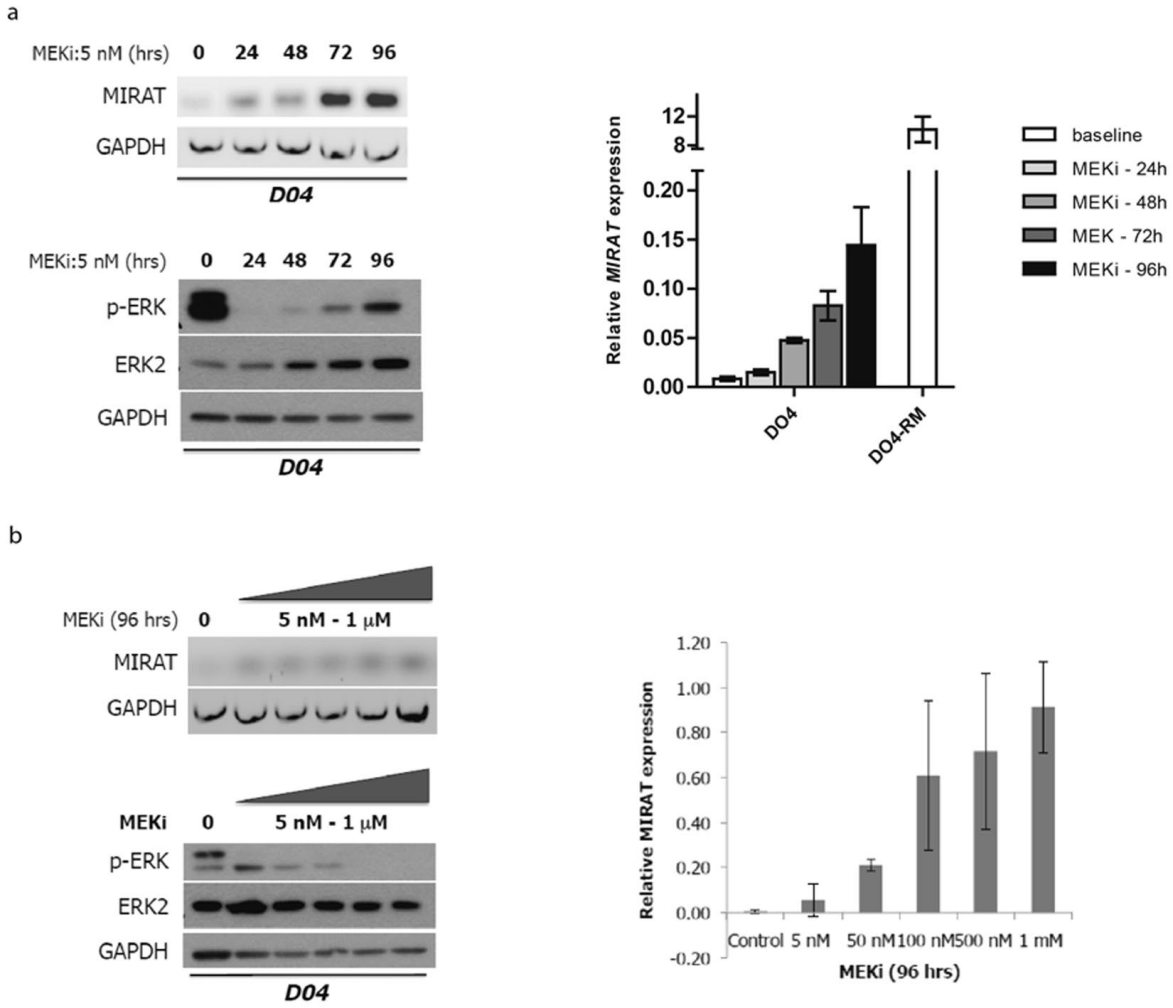
MIRAT gene expression increases in a time and dose dependent manner. (**a**) MIRAT is upregulated under MEK inhibitor treatment with trametinib and increases over time. PCR (top left) and RT-qPCR analyses (top right) of MIRAT in D04 cells treated with 5 nM of the MEK inhibitor at indicated time points (0, 24, 48, 72 and 96 hours). RT-qPCR data is presented as the relative MIRAT expression normalized to GAPDH. Western blot analyses displaying signaling changes in D04 parental, drug-sensitive cells after MEK inhibitor treatment over time: p-ERK levels are reduced and recover over time; (**b**) Increasing doses of the MEK inhibitor trametinib correlate with MIRAT expression. PCR (left) and RT-qPCR analysis (right) of MIRAT were performed on cDNA from D04 cells with different concentrations of the MEK inhibitor (5 nM–1 mM) for 96 hours (Right). Western blot analyses displaying signaling changes in D04 parental, drug-sensitive cells after MEK inhibitor treatment at different doses. Cells show a dose dependent reduction of p-ERK.

Our RNA-Seq experiments detected *MIRAT* in drug-resistant melanoma cell lines but not in those treated for only 6 hours (DO4A, MM415A). To investigate the dynamics of *MIRATs’* expression during time on drug the, we incubated cells with the MEK inhibitor trametinib and quantified *MIRAT* by RT-qPCR at different time points. *MIRAT* was upregulated as early as 24 hours after MEK inhibitor treatment, but at expression levels significantly lower than in resistant clones. Additionally, we found that *MIRAT* expression increased with higher trametinib doses (Fig. 2b). In parallel we tested the effect of the MEK inhibitor trametinib on the MAPK pathway and found a bimodal response to the drug with a p-ERK reduction during early drug incubation an its increase after 96 hours (Fig. 2a,b).

### *MIRAT* could modulate the MAPK signaling pathway

Since *MIRAT* was expressed as early as 24 h after trametinib incubation and increased up to 500-fold in resistant clones (Fig. 2a), we investigated if *MIRAT* was involved in the modulation of the MAPK pathway signaling using transient gene over-expression and RNA-silencing assays.

**Figure 3:**
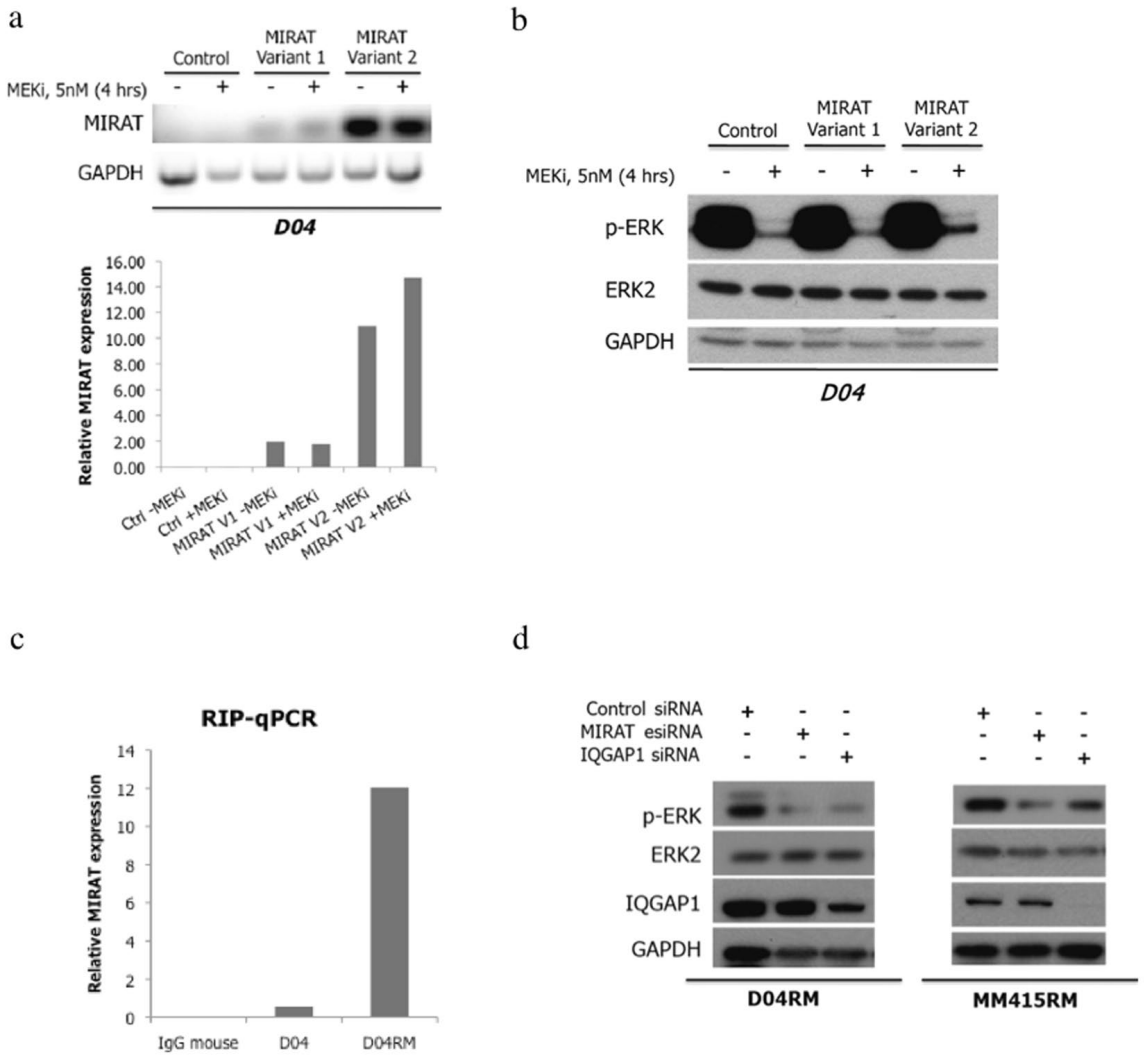
MIRAT modulates MAPK signaling through IQGAP1 binding. (**a**) Ectopic expression of two MIRAT isoforms in D04 parental cells 48 hours post-transfection was determined by PCR (top) and RT-qPCR analysis (bottom) normalized to GAPDH housekeeping gene. (**b**) Immunoblot showing that MIRAT isoform 2 overexpression results in higher p-ERK levels in D04 cells after MEK inhibition with trametinib compared to respective controls. (**c**) Bar graph representing MIRAT relative expression in RNA-protein extract of IQGAP1 pull down. MIRAT was found enriched in the extract of DO4-RM and not in the negative control (IgG mouse). (**d**) Immunoblot showing that silencing of MIRAT and IQGAP1 by esiRNA results in lower p-ERK levels in MM415RM and D04RM trametinib-resistant cells.

We transfected the DO4 melanoma cell line with the two most abundant *MIRAT* isoforms (isoforms 1 and 2). Forty-eight hours after transfection we measured ectopic *MIRAT* levels similar to those observed in DO4RM cells, and confirmed a prevalent cytoplasmatic location of the transcript (Supplementary Fig. 5). The ectopic MIRAT overexpression led to a relative signaling resistance to the MEK inhibitor trametinib, evidenced by higher phospho-ERK levels after trametinib exposure in transfected cells (Fig. 3a,b).

To test whether *MIRAT* silencing might increase signaling sensitivity to MAPK pathway inhibition, we used two independent RNA interference methods: a pool of customized siRNA and the endoribonuclease-prepared siRNAs (esiRNA). esiRNA, known to have less off-target effects than siRNA and to be particularly efficient in silencing noncoding transcripts^29^, was superior in depleting *MIRAT* compared to siRNA and was thus used for all further knockdown experiments (Supplementary Fig. 6). *MIRAT* depletion did not significantly affect cell viability in resistant cells (Supplementary Fig. 7) but led to reduced p-ERK levels in both resistant cell lines tested (Fig. 3c), while the DO4 cells were only minimally affected (Supplementary Fig. 8).

To assess signaling changes in response to MIRAT depletion in resistant cells, we performed gene expression analyses comparing cells treated with esiRNA targeting *MIRAT* to cells treated with control siRNA (Supplementary Dataset 1). ‘*Cell surface receptor linked signal transduction’* was found to be the most affected biological process following *MIRAT* knock-down, supporting its involvement in MAPK related signaling events (Supplementary Fig. 9).

### The effect of *MIRAT* on the MAPK signaling cascade could be mediated by IQGAP1

After we established *MIRAT’s* connection to receptor signaling and the MAPK pathway we aimed to identify its possible binding partners to elucidate molecular mechanisms by which MIRAT interconnects with the MAPK pathway. We used RNA Antisense Purification Mass-Spectrometry (RAP-MS) and RNA binding-protein immunoprecipitation (RIP) to search for possible binding partners. Among possible binding candidates we focused on IQGAP1 (Supplementary Fig. 10, Supplementary Dataset 2). IQGAP1 is a scaffold protein that binds to BRAF, MEK and ERK^30^ and is known to modulate their activity^31^. When we performed RIP experiments we found MIRAT enriched in the IQGAP1 immunoprecipitated products. As MIRAT was found in the negative control IgG-rabbit antibody conditions and in the SNRNP70 antibody (Rabbit polyclonal IgG) pull down (Supplementary Fig. 11).

Binding of IQGAP1 to *MIRAT* was confirmed by enriched *MIRAT* levels in RNA-protein extracts from IQGAP1 pull down experiments, but not in the immunoglobulin G (IgG)-mouse bound sample used as negative control (Fig. 3c). However, *MIRAT* was also found in the negative control IgG-rabbit antibody conditions and in the SNRNP70 antibody (Rabbit polyclonal IgG) pull down (Supplementary Fig. 11).

To verify if *MIRAT* and IQGAP1 are involved in modulating the MAPK pathway, we performed further gene silencing experiments. Knock-down of both, *MIRAT* and IQGAP1, led to decreased levels of p-ERK in both resistant cell lines (Fig. 3d).

## Discussion

The studies of mechanisms of resistance to targeted small molecule inhibitors could lead to a better understanding of tumor biology and thus improve therapeutic strategies. Different mechanisms of resistance as a response to MAPK inhibition have already been reported^13,16,32–35^, but only few studies have assessed the role of lncRNAs^20^. Here, we test how melanoma cell transcriptomes change following MAPK inhibitor treatment. We compare treatment naïve, short-term treated, chronically drug exposed and drug resistant cell lines and identify the lncRNA *MIRAT* which seems to be implicated in MAPK signaling after targeted pathway inhibition.

*MIRAT* mapped to the human chromosome 8q.24.12, a region characterized by only a few protein-coding and several lncRNA genes. This region is known for multiple SNPs and genomic alterations associated with cancer and has emerged as an important region for genetic susceptibility to various malignancies such as breast, prostate, bladder, colon, lung, ovaries, pancreas and brain cancer^36^. Our findings support the biological relevance and indicate that the region 8q.24 is associated with drug resistance in cancer. Although *MIRAT* has recently been annotated, its function and intracellular location has yet not been studied^19^.

We found that *MIRAT* is predominately present in the cytoplasm, which is in contrast to most other known lncRNAs^37^. *MIRAT* silencing and overexpression, as well as RNA-protein interaction results, indicate that this lncRNA modulates the MAPK signaling pathway, at least in the tested NRAS mutant melanoma cell lines. However, this modulation is not sufficient to account for large changes in cell viability and thus is only a part of multiple mechanisms of resistance following small molecule inhibitor treatment. The exact description of such mechanisms of resistance on genetic, epigenetic and protein levels is beyond the scope of this manuscript but further studies are warranted.

Our findings suggest that *MIRAT* functions by binding to the scaffold protein IQGAP1, which is an essential protein facilitating signaling from MEK to ERK^30,31^. IQGAP1 is known to be elevated in various cancers, and could serve as a potential therapeutic target, not only in melanoma but also in other cancers with hyper-activated MAPK signaling^31,38–40^. *MIRAT* could bind to IQGAP1 and stabilize it, decelerate its decay and thus facilitate signaling through the MAPK pathway. Also the fact that *MIRAT* is elevated only 24 hours after MAPK inhibition, points towards a regulatory feedback mechanism.

Interestingly the microarray data shows mostly upregulated genes following *MIRAT’s* knockdown in both cell lines tested. This may be due to a variety of other regulatory mechanisms which follow slight changes in MAPK signaling or due to further still not discovered functions of *MIRAT*. Furthermore, we have to consider that silencing RNA off-target effects cannot be fully excluded.

Taken together, we describe a noncoding RNA directly involved but not leading to drug resistance to small molecule inhibitors. Our results highlight the relevance of cytoplasmic lncRNA for oncogenic signaling pathways, and suggest that *MIRAT* and its protein-binding partner IQGAP1 may serve as potential therapeutic targets in melanoma through their modulation of the MAPK pathway. Future strategies of improving target therapies in cancer are likely to involve selective interference with lincRNAs and their protein-binding partners.

## Material and Methods

### Cell culture and establishment of resistant clones

Human NRAS mutant DO4 and MM415 were provided by Boris Bastian, University of San Francisco, California. DO4 and MM415 harbor NRASQ61L mutation. Cell lines were maintained in RPMI 1640 media (UCSF Cell Culture Facility) supplemented with 10% (vol/vol) heat inactivated fetal bovine serum. Primary avatar cell lines (AV1, AV4, AV5) were obtained from metastases of BRAF mutant patients. All experimental protocols were approved by UCSF Human Research Protection Program Institutional Review Board (IRB# 12-0948), all patients signed informed consent, and methods were carried out in accordance with relevant guidelines and regulations. Cell lines were incubated at 37 °C under 5% CO_2_.

To establish resistant cell lines, NRAS and BRAF mutant cell lines were continuously exposed to increasing concentrations of respectively MEK inhibitor (GSK1120212) (Selleckchem) and BRAF inhibitor PLX-4720 (Selleckchem). Resistant clones were selected, maintained and propagated in a complete medium supplemented with drugs for 6 months. At the end of this time period, the drug sensitivity of the resulting clones was tested seeding cells in 96 well plates with a density of 3,000-5,000 cells per well, treating them with the respective inhibitor increasing concentrations. Cell viability was measured using CellTiter-Glo Luminiscent Cell Viability Assay (Promega; G7570), according to the manufacturer’s protocol. Luminescence was measured on the SynergyHT plate reader (BioTek) using Gen5 software (Version 1.11.5). All experiments were performed at least in triplicates. Cell lines were defined resistant to a drug if GI50 values (concentrations of drugs resulting in 50% growth inhibition relative to controls) were increased by at least two-fold (calculated by CalcuSyn software (Biosoft, Cambridge, UK; Version 2.1)). Resistant clones were termed RM (**R**esistant to **M**APKi): DO4-RM, MM415-RM, AV1-RM, and AV4-RM. AV5 cells did not acquire resistance, therefore the resulting clone after 6 months’ exposure to PLX-4720 was termed ChrExp (**Chr**onical **Exp**osed)

### Immunoblotting

Cell lysates were prepared in RIPA buffer supplemented with Halt protease and phosphatase inhibitor cocktail (Thermo Scientific). Equal amounts of protein, as measured by BCA protein assay, were resolved in 4–12% Bis-Tris NuPage gradient gels (Life Technologies) and transferred electrophoretically on a polyvinylidene difluoride 0.45-m membrane. Membranes were blocked for 1 h at room temperature in 5% bovine serum albumin (BSA) or non-fat milk in TBST before being incubated overnight at 4 °C with the primary antibodies. All primary antibodies were diluted 1:1,000 in 5% BSA or non-fat milk in TBST. After three washes of 5 min in TBST, secondary antibodies were diluted 1:3,000 in 5% non-fat milk in TBST and incubated for 1 h at room temperature. After another three washes in TBST, detection of the signal was achieved by incubating the membrane and exposing it on autoradiography films from Denville Scientific (Metuchen, NJ, USA). The primary antibodies used were: anti-phospho-p44/42 MAPK (Erk1/2) (Thr202/Tyr204) (no. 9101), anti-AKT (no. 9272), anti-phospho-AKT (Ser473) (no. 9271). The secondary antibodies: Amersham ECL anti-rabbit IgG, HRP-linked whole ab from donkey (no. NA934-1ML) and anti-mouse IgG, HRP-linked whole ab from donkey (no. NA931-1ML) were purchased from GE Healthcare Life Sciences. Anti-ERK2 (C-14) (sc-154) was purchased from Santa Cruz Biotechnology. Anti-IQGAP1 (clone AF4) (no. 05-504) was purchased from EMD Millipore. Anti-MITF (C5) was purchased from ThermoFisher Scientific (no. MA5-14146).

### Gene expression analysis

Total RNA was extracted using the miRNeasy Mini Kit (Qiagen, Hilden, Germany). RNA-Seq was performed for 12 cell lines and conditions: DO4, MM415, AV1, AV4, AV5 parental cell lines; DO4 and MM415 cells after short-term (6 hours) treatment with GSK1120212; DO4-RM, MM415-RM, AV1-RM, AV4-RM, and AV5 ChrExp. cDNA sequencing libraries were prepared using the Illumina TruSeq Total RNA Sample kit, which uses Ribo-Zero to deplete rRNA, leaving ncRNAs intact. Paired-end, 101-bp sequencing was performed by Centrillion Biosciencies Inc. (CA, US) on an Illumina HiSeq. 2000. Sequence reads are aligned to the human genome (hg19) using TopHat. Cuffdiff (version 2.1.1) was used with the RefSeq annotation (downloaded from UCSC Genome Browser, May 2013) to detect differentially expressed genes. The data have been deposited in NCBI’s Gene Expression Omnibus^41^ and are accessible through GEO Series accession number GSE 99867 (https://www.ncbi.nlm.nih.gov/geo/query/acc.cgi?acc=GSE 99867).

### Patient sample collection and RNA extraction

We selected a set of patient melanoma tumors formalin-fixed paraffin-embedded (FFPE) blocks from the tissue bank of our institution (University of California, San Francisco). All experimental protocols were approved by UCSF Human Research Protection Program Institutional Review Board (IRB# 12-0948), patients signed informed consent, and procedures were carried out in accordance with relevant guidelines and regulations. RNA was extracted using RNeasy FFPE kit (Qiagen, Valencia, CA). RNA yield and A260/A280 ratio were monitored with a NanoDrop ND-1000 spectrometer (NanoDrop Technologies). We pre-amplified the transcripts and performed TaqMan-qRT-PCR. GAPDH was used as an endogenous control, and to express the relative abundance of the transcripts making a comparison between samples possible. Patient information was extracted retrospectively from the electronic patient charts into an excel file, following patient anonymization. The TANRIC web application was used to test MIRAT expression among available TCGA melanoma samples accordingly to described methods^28^.

### RACE analysis

SMARTer RACE 5′/3′ Kit (no. 634859) was purchased from Clontech Laboratories, Inc. The analysis was performed accordingly to the manufacturer’s protocol. Used sequences were: 5′ RACE sense: GATTACGCCAAGCTTACTGATCCTTGAATGGGTTGCTATAATTACATTTTAAAGAGT (Tm = 65 °C). 3′ RACE sense: GATTACGCCAAGCTTGGTCACCAGGGCTCTTCTTCTCGGA (Tm = 69.2 °C).

### Cloning procedures

MIRAT cloning was designed and synthesized using the high-fidelity DNA polymerase Phusion Hot Start Flex DNA polymerase, and cloned into pcDNA-DEST40 Gateway vector.

### siRNA and esiRNA

For siRNA studies, we designed customized siRNAs against *MIRAT* and used Control siRNA and ON-TARGET plus Human IQGAP1 (8826) siRNA–SMARTpool (004694-00-0005) (Thermo Fisher Scientific, Waltham, USA).

Cell lines were plated in 96 well plates with a density of 4000–8000 cells per well. After 24 hours, cells were transfected at a final concentration of 25 nM of siRNA, using Lipofectamine 2000 (Invitrogen, CA, USA) as per manufacturer’s instructions and incubated for 72 h post transfection. All siRNA experiments were performed at least in triplicates.

For esiRNA, we designed esiRNA libraries as previously described^42,43^. Briefly, esiRNA library was designed using DEQOR (a web based tool for design of siRNAs^44^) choosing the optimal gene-silencing region for PCR (400bp-600bp), primer design, attaching the T7 RNA polymerase promotor to the 5′ end of each primer. esiRNA were synthesized with the following steps: template generation, *in vitro* transcription to generate long dsRNA, digestion to generate a pool of short dsRNAs, purification of esiRNA. Transfection was performed with Lipofectamine.

### RIP assay and RNA antisense purification-mass spectrometry (RAP-MS)

Magna RIP^TM^ RNA-Binding Protein Immunoprecipitation Kit (no. 17–700) was performed to identify and validate the protein-binding partner of MIRAT, accordingly to the manufacturer’s protocol. RNA antisense purification and mass spectrometry were performed as previously described^20,45–48^.

### Subcellular fractionation

Subcellular fractionation and RNA purification for the localization of MIRAT were performed using the Cytoplasmic and nuclear RNA purification Kit (#2100,34700) according to the manufacturer’s protocol.

### Oligonucleotide sequences 5′-3′, probes and primers

*MIRAT I Forward:* CTCGGAGGGCCCATGA and Reverse: GGGAGCAGAGGGGAATG; MIRAT II Forward: CGGAGAAGACAAGACG and Reverse: CCCATGGGCCCCTTAC. *GAPDH Forward:* AGAAGACTGTGGATGGCCCC and Reverse: AGGTCCACCACCCTGTTGC. *MALAT1 Forward:* AGAAGACTGTGGATGGCCCC and Reverse: AGGTCCACCACCCTGTTGC. *MITF Forward:* CGAGCTCATGGACTTTCCCTTA and Reverse: CTTGATGATCCGATTCACCAAA. *MIRAT DNA Biotin-TEG3*′ *probes*: 1-CATGGTGGAGGAAGTCATGG, 2-CTATGAGTCCGGCATTTGAA, 3-CGCAGGATTTAGTCTGGTAG, 4-CATTGTCACACACTCTCCTC, 5-CCTCTGTACTGTGTCATTAC, 6-ACTGATCCTTGAATGGGTTG, 7-AGCTCAGATTTCTCAATGCT, 8-ATTGCGTAGGTATGCGGAAT.

### Microarray analysis

Microarray analysis was performed using the GeneChip Human Gene 2.0 ST Array (902112) (Affymetrix, Thermo Fisher Scientific, Santa Clara, CA, USA) according to the manufacturer’s protocol. For experimental design and data analysis see Supplementary Fig. 5.

### Statistical analyses

The statistical analyses were performed using Graphpad Prism V.7.0c and Stata 12.0 statistical software. Comparisons between two independent non-normally distributed groups were performed using the nonparametric Wilcoxon rank-sum test; P values less than 0.05 were considered statistically significant.

## Electronic supplementary material

Supplementary Figures and Tables

Supplementary Dataset 1

Supplementary Dataset 2
