# Supplementary material for: The lincRNA MIRAT binds to IQGAP1 and modulates the MAPK pathway in NRAS mutant melanoma
Source: Sci Rep. 2018 Jul 19;8:10902. doi: 10.1038/s41598-018-27643-3 (PMC6053443; doi:10.1038/s41598-018-27643-3)
Supplement: Supplementary file 1 — Supplementary Figures and Tables [file 41598_2018_27643_MOESM1_ESM.docx]

**The lincRNA *MIRAT* binds to IQGAP1 and modulates the MAPK pathway in NRAS mutant melanoma**

Martina Sanlorenzo^*1,2,3^, Igor Vujic^*1,4,5^, Rosaura Esteve-Puig^*1^, Kevin Lai^1^, Marin Vujic^1^, Kevin Lin^1^, Christian Posch^1,4,5^, Michelle Dimon^1^, Adrian Moy^1^, Mitchell Zekhtser^1^, Katia Johnston^1^, Deborah Gho^1^, Wilson Ho^1^, Abhinay Gajjala, ^1^ Juan Oses-Prieto^6^, Alma Burlingame^6^, Adil Daud^7^, Klemens Rappersberger^2,5^, Susana Ortiz-Urda^1^

^*^ contributed equally

**SUPPLEMENTARY FIGURES**


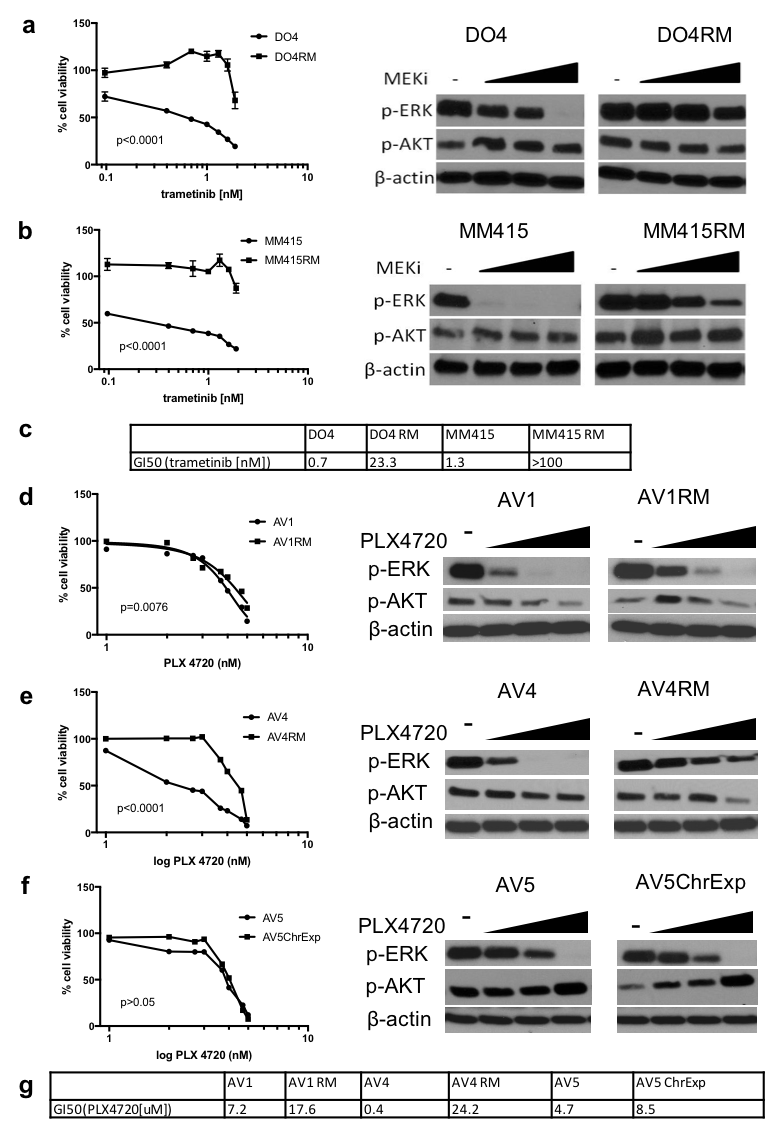


***Supplementary Figure 1: NRAS and BRAF mutant melanoma cells were made resistant respectively to trametinib and PLX 4270 in vitro.*** *Growth curves and immunoblotting of parental and resistant clones exposed to increasing concentration of drugs: (a) DO4. (b) MM415. (d) AV1. (e) AV4. and (f) AV5 (nonlinear regression curve fit, logGI50 values; Graphpad Prism V.7.0c). Tables indicating the GI50 values of the (c) NRAS and (g) BRAF mutant cells (CalcuSyn software; Version 2.1).*


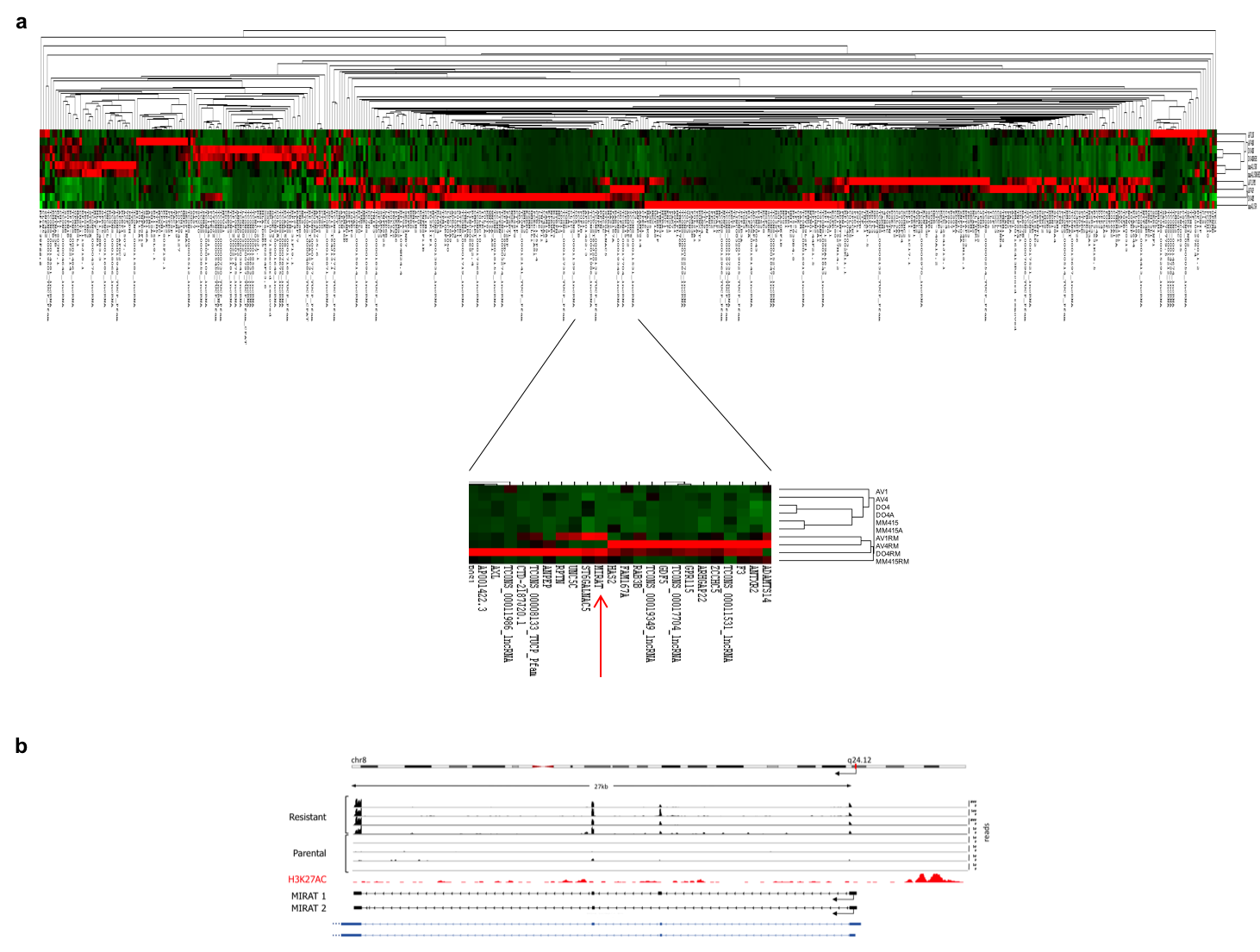


***Supplementary Figure 2: Hierarchical clustering, heatmap, and schematic representation of MIRAT lincRNA.*** *(a) The web-based Morpheus tool was used to create the heatmap (Parameters: Metric=1-pearson correlation). Hierarchical clustering was performed using the differential expressed genes found in each cell line. Zoom-in to the area where the lincRNA MIRAT was found. (b) MIRAT gene locus spans a ~27kb region on chromosome 8q24.12. RNA-Seq reads for MIRAT are shown in resistant and parental melanoma cell lines respectively (top to bottom: D04. MM415. AV1. AV4). H3K27AC ChIP-Seq peaks are depicted as important indicator of enhancer activity or transcriptionally active promoter (red). highest expressed MIRAT isoforms (MIRAT 1 and MIRAT 2) assembled in Cufflinks/Scripture (black). and representative partial isoforms from MiTranscriptome (blue).*

*
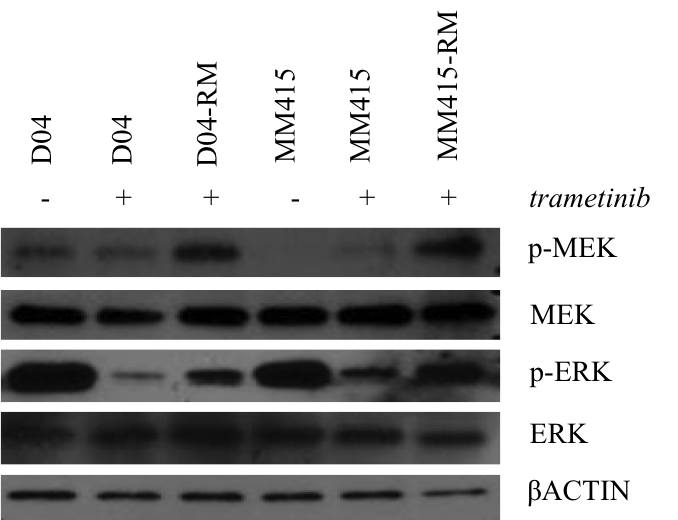
*

***Supplementary Figure 3: MEK protein levels were comparable between NRAS mutant parental and resistant cell.*** *Immunoblot analyses for effector proteins of the MAPK pathway. Trametinib effectively reduces p-ERK levels in parental cell lines. Resistant cells with the same concentration of trametinib show higher p-ERK and p-MEK levels.*

*
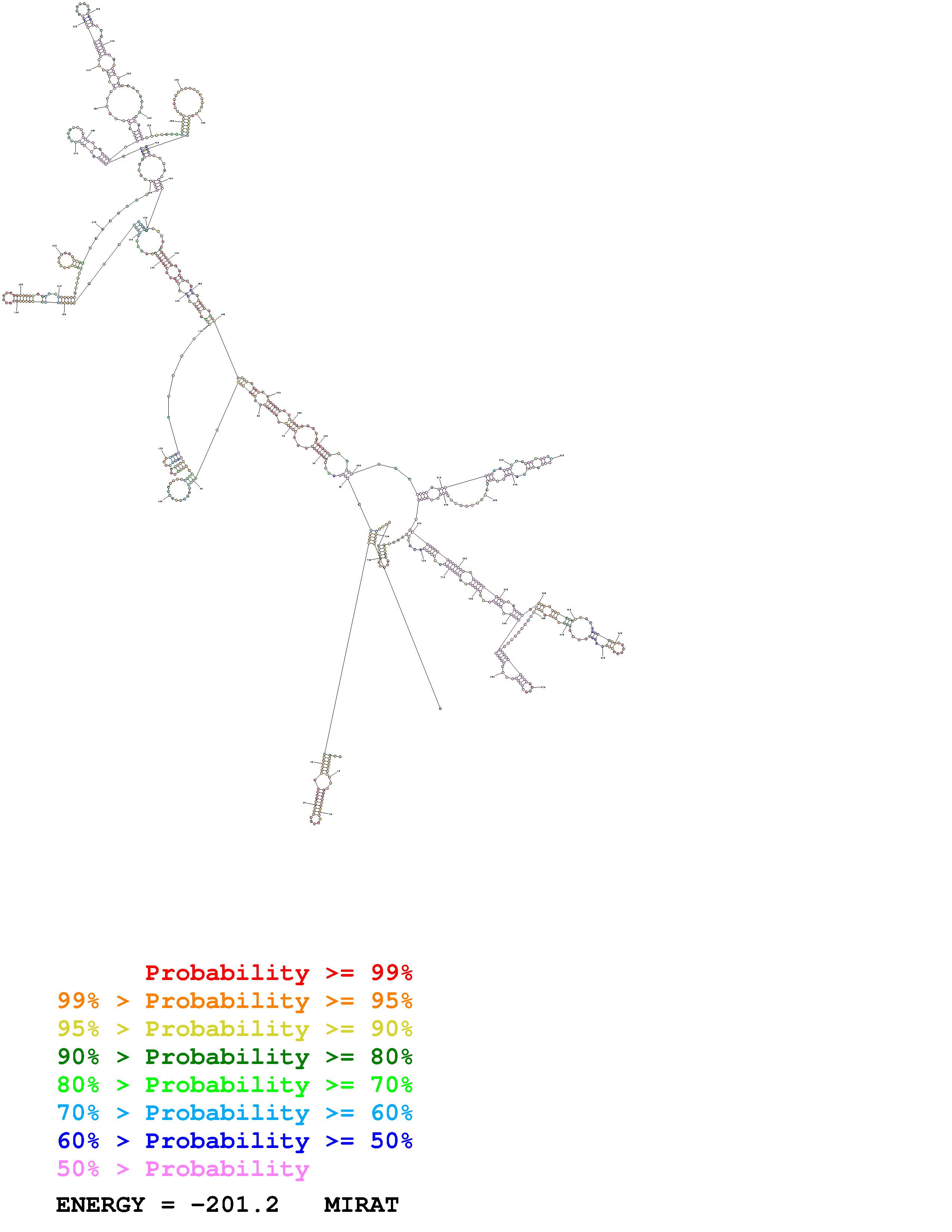
*

***Supplementary Figure 4: MIRAT secondary structure.*** *MIRAT secondary structure was predicted using RNAstructure software (Mathews Lab, Department of Biochemistry & Biophysics, University of Rochester Medical Center)with default setting.*


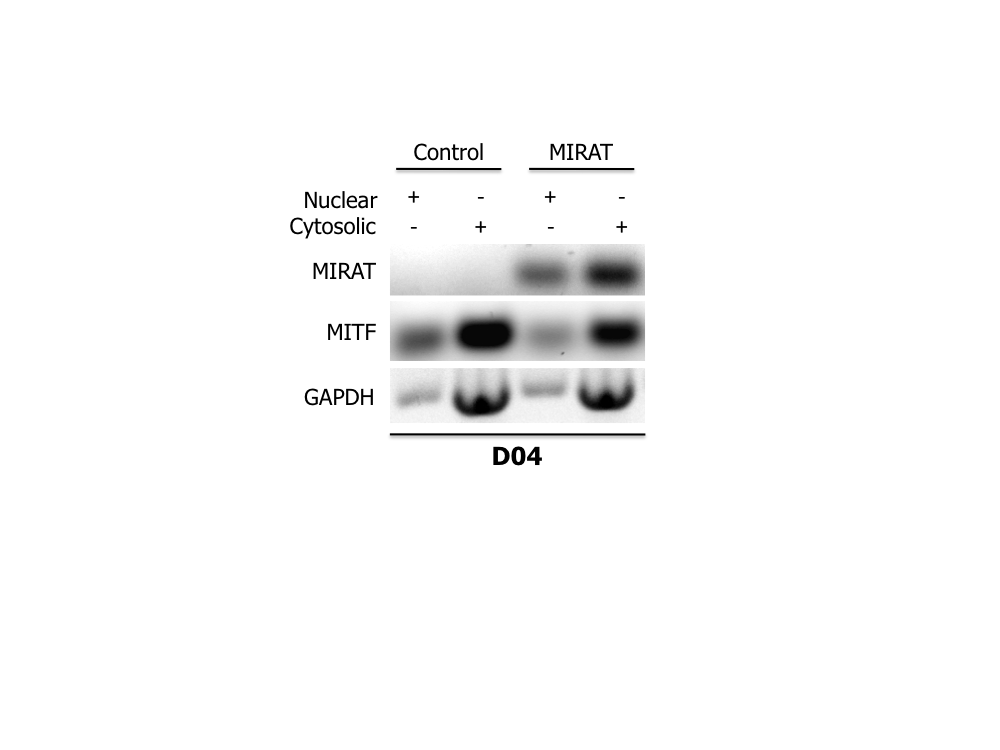


***Supplementary Figure 5:* *Ectopic MIRAT localized in the cytosolic compartment.*** *Standard agarose gel electrophoresis of MIRAT was performed on nuclear and cytosolic extraction from DO4 cells. MITF was used as a cytosolic control and GAPDH as a loading control.*

***Supplementary Fig. 6: endoribonuclease-prepared siRNAs esiRNA is superior to siRNA in silencing MIRAT.*** *Bar graph depicturing the relative MIRAT expression to GAPDH in at least triplicates.*

*
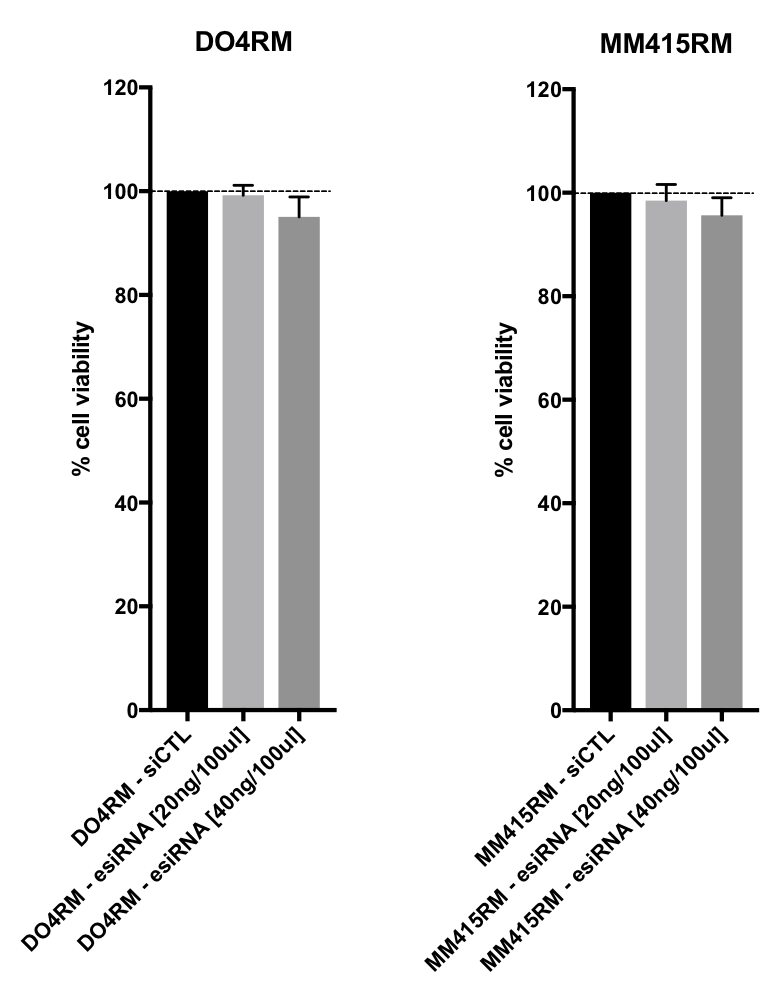
*

***Supplementary Fig. 7: MIRAT esiRNA-silencing did not affect cell viability in resistant cell lines.*** *Bar graph depicturing cell viability compared to siCTL in resistant cell lines (DO4RM and MM415RM) treated with 20ng/100ul and 40ng/100ul of esiRNA for 96 hours.*

*
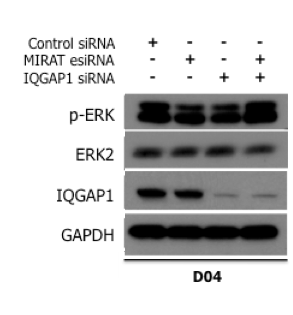
*

***Supplementary Fig. 8: MIRAT esiRNA-silencing only minimally affected p-ERK levels in DO4 parental cell lines.*** *Immunoblot of parental DO4 cell lines treated with control siRNA, MIRAT esiRNA, and IQGAP1 siRNA.*


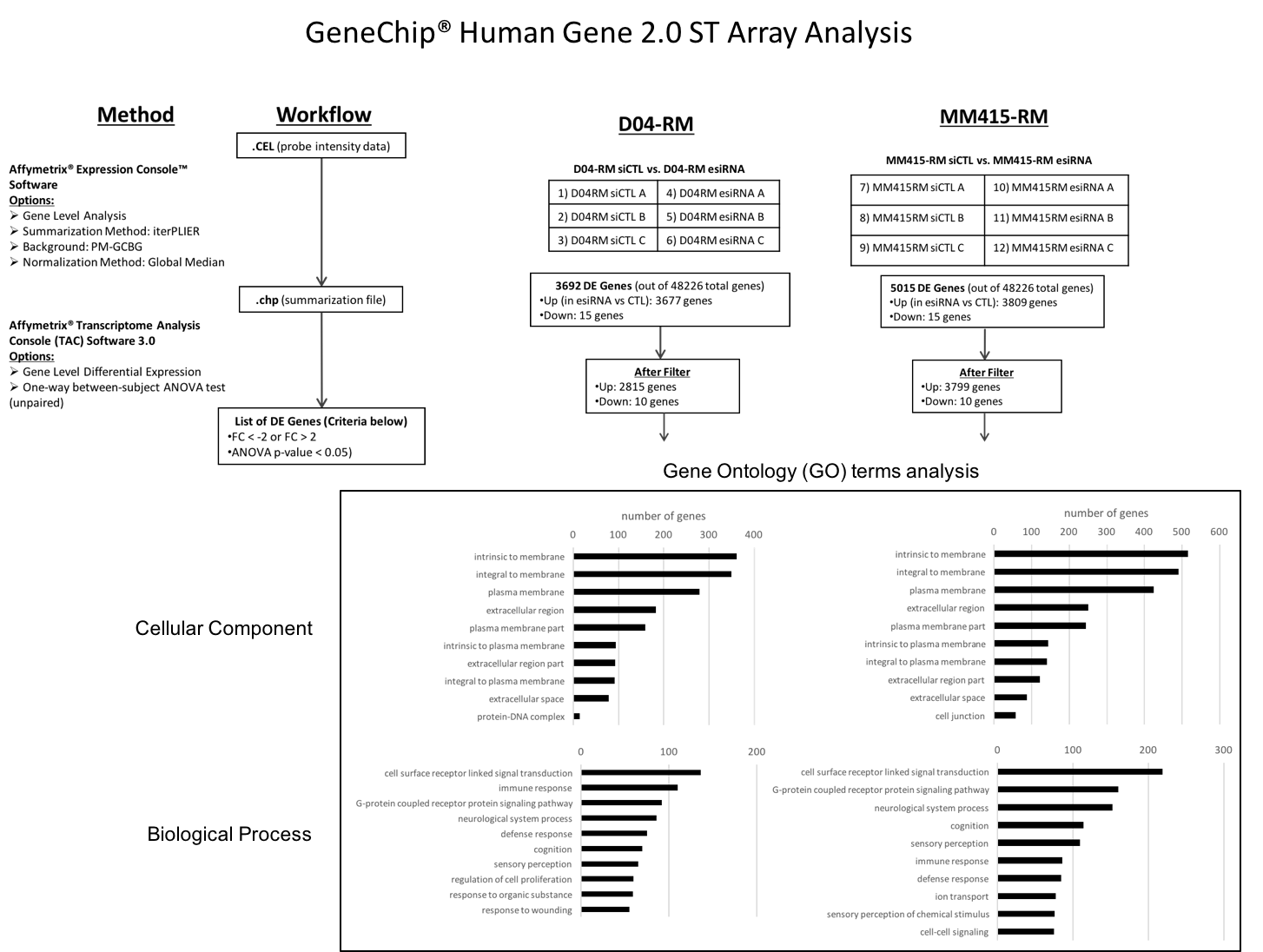


***Supplementary Fig. 9: MIRAT silencing impacts the signal transduction from cell surface receptors.*** *RNA extracts after confirmed esiRNA based MIRAT depletion were analyzed and compared to siCTL treated controls (n=3 for each cell line) using the GeneChip Human Gene 2.0 ST Array. Depictured is the bioinformatic pipeline of the Affimetrix Array analyses and the resulting gene ontology (GO) terms. MIRAT silencing resulted in 2815 upregulated genes in DO4RM and 3799 genes in MM415RM. and in the downregulation of 10 genes in both. DO4RM and MM415RM. GO terms analyses of differently expressed genes in the two cell lines showing GO terms involved in signal transduction from the cell surface receptors.*


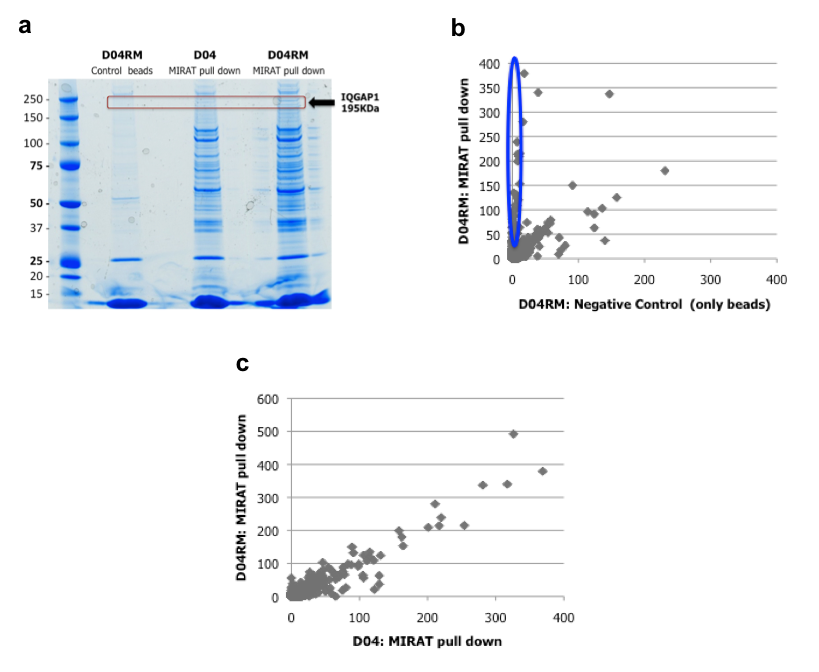


***Supplementary Fig. 10: Interactome studies by RNA Antisense Purification Mass-Spectrometry (RAP-MS) enabled purification of endogenous MIRAT RNA-protein complexes****. Endogenous MIRAT RNA-protein complexes were purified by the RAP-MS methodology. (a) After purification of MIRAT RNA-protein complexes. proteins were separated by gel electrophoresis and stained by Colloidal Coomassie Blue. Lanes (left to right): Protein Ladder. D04RM Negative Control (beads only). D04 pull-down with same probes an internal control to subtract non-specific for MIRAT protein-binding partners. and D04RM MIRAT pull-down to identify potential protein-binding partners associated with drug resistance. (b) The number of peptides identified by MS are represented in a comparison plot between D04RM Negative control (only beads) and D04RM MIRAT pull-down. Blue oval contains the specific MIRAT protein-binding partners with the highest peptide values. (c) The diagonal line on the graph comparing peptide numbers between D04 parental MIRAT pull-down and D04RM drug-resistant MIRAT pull-down represents putative MIRAT protein-binding partner as well as non-specific nucleic acid binding proteins. Proteins with differential abundances between DO4RM/DO4 represent candidates associated to drug resistance.*

*
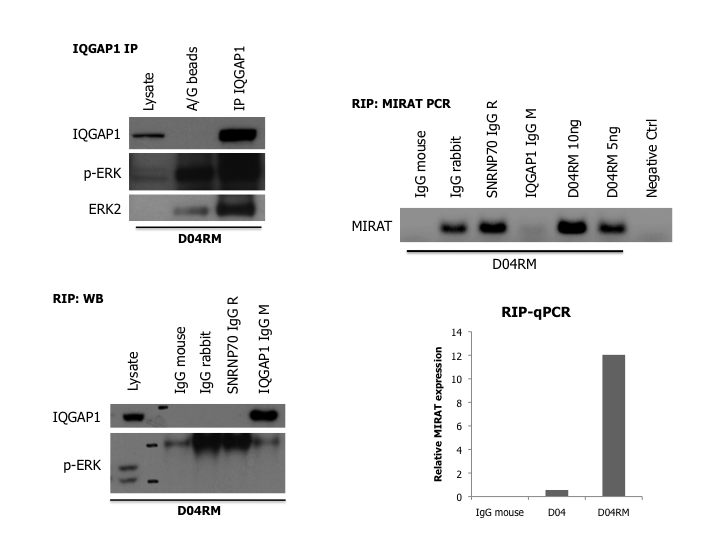
*

***Supplementary Fig. 11: IQGAP1 pull down.*** *Standard agarose gel electrophoresis of MIRAT-PCR products. MIRAT was detected in IQGAP1 mouse monoclonal antibody (IQGAP1 IgG M) and not in the control IgG mouse. MIRAT was unspecifically found in the IgG rabbit control and in the SNRNP70 IgG rabbit conditions.*

**SUPPLEMENTARY TABLES**

***Supplementary Table 1:*** *Genes differentially expressed in DO4-RM compared to DO4. after filtering for genes differently expressed in DO4-A*

| Names of Transcripts | Category | sample_1 | sample_2 | value_1 | value_2 | q_value |
| --- | --- | --- | --- | --- | --- | --- |
| CDA | ProteinCoding | D04 | DO4-RM | 0 | 649.096 | 0,0090385 |
| TCONS_00002181_lncRNA | Novel lncRNA | D04 | DO4-RM | 406.738 | 0 | 0,0090385 |
| LPHN2 | ProteinCoding | D04 | DO4-RM | 0 | 283.025 | 0,0090385 |
| NTNG1 | ProteinCoding | D04 | DO4-RM | 0 | 133.681 | 0,0090385 |
| TCONS_00004376_lncRNA | Novel lncRNA | D04 | DO4-RM | 0,499812 | 0,0531245 | 0,0090385 |
| LGR6 | ProteinCoding | D04 | DO4-RM | 0 | 579.724 | 0,0090385 |
| IL24 | ProteinCoding | D04 | DO4-RM | 321,3 | 426.435 | 0,0090385 |
| TRIM63 | ProteinCoding | D04 | DO4-RM | 727.026 | 0 | 0,0090385 |
| TCONS_00011393_lncRNA | Novel lncRNA | D04 | DO4-RM | 0 | 428.684 | 0,0090385 |
| TCONS_00011843_lncRNA | Novel lncRNA | D04 | DO4-RM | 0 | 0,846045 | 0,0090385 |
| TCONS_00012735_TUCP_Pfam | TUCP | D04 | DO4-RM | 0 | 699.945 | 0,0090385 |
| TCONS_00012940_TUCP_Pfam | Novel lncRNA | D04 | DO4-RM | 0,584449 | 0 | 0,0090385 |
| COL11A1 | ProteinCoding | D04 | DO4-RM | 112.171 | 926.275 | 0,0090385 |
| TCONS_00013834_lncRNA | Novel lncRNA | D04 | DO4-RM | 0 | 672.149 | 0,0090385 |
| TCONS_00014581_lncRNA | Novel lncRNA | D04 | DO4-RM | 100.687 | 0 | 0,0090385 |
| TCONS_00014865_lncRNA | Novel lncRNA | D04 | DO4-RM | 444.439 | 0,342138 | 0,0090385 |
| CHI3L1 | ProteinCoding | D04 | DO4-RM | 0 | 552.865 | 0,0090385 |
| ESRRG | ProteinCoding | D04 | DO4-RM | 0 | 0,872424 | 0,0090385 |
| TCONS_00017166_TUCP_Pfam | Novel lncRNA | D04 | DO4-RM | 171.111 | 799.443 | 0,0090385 |
| TCONS_00017368_lncRNA | Novel lncRNA | D04 | DO4-RM | 0 | 155.178 | 0,0090385 |
| TCONS_00017886_lncRNA | Novel lncRNA | D04 | DO4-RM | 312.141 | 0,770668 | 0,0090385 |
| **MIRAT** | Novel lncRNA | D04 | DO4-RM | 0 | 169.347 | 0,0090385 |
| TCONS_00018633_lncRNA | Novel lncRNA | D04 | DO4-RM | 327.235 | 0,00671624 | 0,0090385 |
| PLAU | ProteinCoding | D04 | DO4-RM | 0 | 135.388 | 0,0090385 |
| TCONS_00019475_lncRNA | Novel lncRNA | D04 | DO4-RM | 175.178 | 0 | 0,0090385 |
| ARMC4 | ProteinCoding | D04 | DO4-RM | 0 | 0,59532 | 0,0090385 |
| SLC16A9 | ProteinCoding | D04 | DO4-RM | 0 | 0,598584 | 0,0090385 |
| KIAA1598 | ProteinCoding | D04 | DO4-RM | 83.335 | 424.754 | 0,0090385 |
| RP11-20J1.1 | Known_lncRNA | D04 | DO4-RM | 433.379 | 0 | 0,0090385 |
| RP11-27G22.1 | Known_lncRNA | D04 | DO4-RM | 0,947237 | 0 | 0,0090385 |
| MMP13 | ProteinCoding | D04 | DO4-RM | 0 | 246.652 | 0,0090385 |
| PCED1B | ProteinCoding | D04 | DO4-RM | 0 | 14.037 | 0,0090385 |
| ERBB3 | ProteinCoding | D04 | DO4-RM | 933.347 | 632.579 | 0,0090385 |
| SLC38A4 | ProteinCoding | D04 | DO4-RM | 0 | 391.601 | 0,0090385 |
| PCED1B-AS1 | Known_lncRNA | D04 | DO4-RM | 0 | 620.183 | 0,0090385 |
| TMEM119 | ProteinCoding | D04 | DO4-RM | 0 | 441.209 | 0,0090385 |
| LINC00332 | Known_lncRNA | D04 | DO4-RM | 0,827507 | 0 | 0,0090385 |
| TNFSF11 | ProteinCoding | D04 | DO4-RM | 0 | 28.878 | 0,0090385 |
| POSTN | ProteinCoding | D04 | DO4-RM | 0 | 134.093 | 0,0090385 |
| TRPC4 | ProteinCoding | D04 | DO4-RM | 0 | 341.702 | 0,0090385 |
| LINC01050 | Known_lncRNA | D04 | DO4-RM | 0 | 274.245 | 0,0090385 |
| ITGA11 | ProteinCoding | D04 | DO4-RM | 0 | 190.964 | 0,0090385 |
| MMP2 | ProteinCoding | D04 | DO4-RM | 133.652 | 216.102 | 0,0090385 |
| RP11-80F22.14 | Known_lncRNA | D04 | DO4-RM | 282.171 | 0 | 0,0090385 |
| CDH11 | ProteinCoding | D04 | DO4-RM | 0 | 909.951 | 0,0090385 |
| ITGA3 | ProteinCoding | D04 | DO4-RM | 691.151 | 191.888 | 0,0090385 |
| RTN4RL1 | ProteinCoding | D04 | DO4-RM | 0 | 176.924 | 0,0090385 |
| RP11-1094H24.4 | Known_lncRNA | D04 | DO4-RM | 0 | 148.996 | 0,0090385 |
| COLEC12 | ProteinCoding | D04 | DO4-RM | 0 | 338.153 | 0,0090385 |
| UCA1 | Known_lncRNA | D04 | DO4-RM | 0 | 40.171 | 0,0090385 |
| F2RL3 | ProteinCoding | D04 | DO4-RM | 0 | 558.733 | 0,0090385 |
| TEX41 | Known_lncRNA | D04 | DO4-RM | 108.764 | 0,591495 | 0,0090385 |
| ZNF804A | ProteinCoding | D04 | DO4-RM | 0 | 159.606 | 0,0090385 |
| CYTIP | ProteinCoding | D04 | DO4-RM | 0 | 567.525 | 0,0090385 |
| NGEF | ProteinCoding | D04 | DO4-RM | 0 | 259.239 | 0,0090385 |
| TGM2 | ProteinCoding | D04 | DO4-RM | 779.304 | 263.169 | 0,0090385 |
| JPH2 | ProteinCoding | D04 | DO4-RM | 0 | 0,758582 | 0,0090385 |
| ANKRD30BP2 | Known_lncRNA | D04 | DO4-RM | 0,764442 | 0 | 0,0090385 |
| COL6A1 | ProteinCoding | D04 | DO4-RM | 493.509 | 127,09 | 0,0090385 |
| COL6A2 | ProteinCoding | D04 | DO4-RM | 578.916 | 250.996 | 0,0090385 |
| AP001422.3 | Known_lncRNA | D04 | DO4-RM | 0 | 0,716349 | 0,0090385 |
| S100B | ProteinCoding | D04 | DO4-RM | 573.271 | 150.353 | 0,0090385 |
| SOX10 | ProteinCoding | D04 | DO4-RM | 64.019 | 230.997 | 0,0090385 |
| STAC | ProteinCoding | D04 | DO4-RM | 0 | 320.969 | 0,0090385 |
| MME | ProteinCoding | D04 | DO4-RM | 0 | 145.188 | 0,0090385 |
| RP11-889D3.1 | Known_lncRNA | D04 | DO4-RM | 0 | 0,962169 | 0,0090385 |
| WNT5A | ProteinCoding | D04 | DO4-RM | 0 | 218,76 | 0,0090385 |
| FAM19A4 | ProteinCoding | D04 | DO4-RM | 0 | 430.787 | 0,0090385 |
| MUC13 | ProteinCoding | D04 | DO4-RM | 0 | 5.261 | 0,0090385 |
| RP11-807H7.1 | Known_lncRNA | D04 | DO4-RM | 0 | 145.329 | 0,0090385 |
| TMEM156 | ProteinCoding | D04 | DO4-RM | 0 | 450.085 | 0,0090385 |
| ANTXR2 | ProteinCoding | D04 | DO4-RM | 0,652944 | 294.154 | 0,0090385 |
| TNIP3 | ProteinCoding | D04 | DO4-RM | 0 | 163.579 | 0,0090385 |
| RP11-119J18.1 | Known_lncRNA | D04 | DO4-RM | 0,379481 | 0 | 0,0090385 |
| TGFBI | ProteinCoding | D04 | DO4-RM | 649.167 | 576.251 | 0,0090385 |
| SEPP1 | ProteinCoding | D04 | DO4-RM | 0 | 134.146 | 0,0090385 |
| EDIL3 | ProteinCoding | D04 | DO4-RM | 0 | 465.464 | 0,0090385 |
| CD74 | ProteinCoding | D04 | DO4-RM | 928.705 | 310.494 | 0,0090385 |
| HLA-DRB1 | ProteinCoding | D04 | DO4-RM | 336.647 | 228.917 | 0,0090385 |
| XXbac-BPG254F23.6 | Known_lncRNA | D04 | DO4-RM | 231.966 | 394.264 | 0,0090385 |
| COL1A2 | ProteinCoding | D04 | DO4-RM | 204.209 | 349.723 | 0,0090385 |
| CAV1 | ProteinCoding | D04 | DO4-RM | 186.134 | 279.648 | 0,0090385 |
| KCND2 | ProteinCoding | D04 | DO4-RM | 0 | 147.542 | 0,0090385 |
| IGFBP3 | ProteinCoding | D04 | DO4-RM | 26.399 | 254.353 | 0,0090385 |
| TSPAN12 | ProteinCoding | D04 | DO4-RM | 0 | 279.211 | 0,0090385 |
| SCARA3 | ProteinCoding | D04 | DO4-RM | 0 | 118.841 | 0,0090385 |
| COLEC10 | ProteinCoding | D04 | DO4-RM | 0 | 512.542 | 0,0090385 |
| FAM167A | ProteinCoding | D04 | DO4-RM | 0 | 331.916 | 0,0090385 |
| TNFRSF11B | ProteinCoding | D04 | DO4-RM | 0 | 845.332 | 0,0090385 |
| GDA | ProteinCoding | D04 | DO4-RM | 0 | 275.418 | 0,0090385 |
| CYSRT1 | ProteinCoding | D04 | DO4-RM | 0 | 564.866 | 0,0090385 |
| CLIC3 | ProteinCoding | D04 | DO4-RM | 0 | 102.259 | 0,0090385 |
| SSX1 | ProteinCoding | D04 | DO4-RM | 973.347 | 0 | 0,0090385 |
| GAGE13 | ProteinCoding | D04 | DO4-RM | 135.442 | 0 | 0,0090385 |
| PLP1 | ProteinCoding | D04 | DO4-RM | 337.762 | 983.849 | 0,0090385 |
| CT45A2 | ProteinCoding | D04 | DO4-RM | 0 | 215.291 | 0,0090385 |
| POF1B | ProteinCoding | D04 | DO4-RM | 0 | 157.249 | 0,0090385 |
| TCONS_00000797_TUCP_Pfam_CPAT | TUCP | D04 | DO4-RM | 240.588 | 0 | 0,0145782 |
| TCONS_00001096_lncRNA | Novel lncRNA | D04 | DO4-RM | 0,912097 | 0 | 0,0145782 |
| TCONS_00003274_lncRNA | Novel lncRNA | D04 | DO4-RM | 0 | 14.782 | 0,0145782 |
| TCONS_00008132_TUCP_Pfam | Novel lncRNA | D04 | DO4-RM | 0 | 114.504 | 0,0145782 |
| TCONS_00008133_TUCP_Pfam | TUCP | D04 | DO4-RM | 0 | 101.811 | 0,0145782 |
| TCONS_00007890_lncRNA | Novel lncRNA | D04 | DO4-RM | 121.812 | 0 | 0,0145782 |
| MYBPH | ProteinCoding | D04 | DO4-RM | 0 | 225.401 | 0,0145782 |
| PLEKHA6 | ProteinCoding | D04 | DO4-RM | 0 | 0,615405 | 0,0145782 |
| TCONS_00019349_lncRNA | Novel lncRNA | D04 | DO4-RM | 0 | 0,585163 | 0,0145782 |
| FRG2DP | Known_lncRNA | D04 | DO4-RM | 128.142 | 0 | 0,0145782 |
| TMEM92 | ProteinCoding | D04 | DO4-RM | 0 | 16.487 | 0,0145782 |
| AXL | ProteinCoding | D04 | DO4-RM | 21.832 | 309.756 | 0,0145782 |
| IGFBP2 | ProteinCoding | D04 | DO4-RM | 202.142 | 0 | 0,0145782 |
| GDF5 | ProteinCoding | D04 | DO4-RM | 0 | 234.138 | 0,0145782 |
| WNT5A-AS1 | Known_lncRNA | D04 | DO4-RM | 0 | 100.694 | 0,0145782 |
| MITF | ProteinCoding | D04 | DO4-RM | 128.373 | 439.567 | 0,0145782 |
| EPHB1 | ProteinCoding | D04 | DO4-RM | 0 | 117.981 | 0,0145782 |
| PARM1 | ProteinCoding | D04 | DO4-RM | 0 | 0,46715 | 0,0145782 |
| IL31RA | ProteinCoding | D04 | DO4-RM | 0 | 279.816 | 0,0145782 |
| HLA-DRB5 | ProteinCoding | D04 | DO4-RM | 276.853 | 802.906 | 0,0145782 |
| GPNMB | ProteinCoding | D04 | DO4-RM | 249.181 | 241.011 | 0,0145782 |
| NR_003715 | Known_lncRNA | D04 | DO4-RM | 149.028 | 0 | 0,0145782 |
| RP11-1002K11.1 | Known_lncRNA | D04 | DO4-RM | 0 | 312.354 | 0,0145782 |
| TCONS_00001719_lncRNA | Novel lncRNA | D04 | DO4-RM | 0 | 319.834 | 0,0203876 |
| TCONS_00004648_lncRNA | Novel lncRNA | D04 | DO4-RM | 178.765 | 0 | 0,0203876 |
| TCONS_00018155_lncRNA | Novel lncRNA | D04 | DO4-RM | 0 | 479.502 | 0,0203876 |
| LINC00297 | Known_lncRNA | D04 | DO4-RM | 0,455502 | 0 | 0,0203876 |
| MT1E | ProteinCoding | D04 | DO4-RM | 117.718 | 0 | 0,0203876 |
| MIR146A | Known_lncRNA | D04 | DO4-RM | 126.277 | 0,496436 | 0,0203876 |
| COL12A1 | ProteinCoding | D04 | DO4-RM | 686.956 | 552.258 | 0,0203876 |
| CLU | Known_lncRNA | D04 | DO4-RM | 0,564979 | 586.808 | 0,0203876 |
| GPM6B | ProteinCoding | D04 | DO4-RM | 110.315 | 280.523 | 0,0203876 |
| TCONS_00006675_lncRNA | Novel lncRNA | D04 | DO4-RM | 829.766 | 0,363604 | 0,0252825 |
| TCONS_00013697_lncRNA | Novel lncRNA | D04 | DO4-RM | 0 | 0,788227 | 0,0252825 |
| TCONS_00017775_TUCP_Pfam | TUCP | D04 | DO4-RM | 172.815 | 0 | 0,0252825 |
| TCONS_00018066_lncRNA | Novel lncRNA | D04 | DO4-RM | 0,641724 | 0 | 0,0252825 |
| RP11-164J13.1 | Known_lncRNA | D04 | DO4-RM | 57.762 | 156.408 | 0,0252825 |
| TPM1 | ProteinCoding | D04 | DO4-RM | 377.471 | 363.167 | 0,0252825 |
| GZMM | ProteinCoding | D04 | DO4-RM | 0 | 530.187 | 0,0252825 |
| GALNT13 | ProteinCoding | D04 | DO4-RM | 0 | 0,485946 | 0,0252825 |
| MTUS1 | ProteinCoding | D04 | DO4-RM | 295.274 | 13.437 | 0,0252825 |
| FGFR1 | ProteinCoding | D04 | DO4-RM | 0 | 0,607757 | 0,0252825 |
| LYST | ProteinCoding | D04 | DO4-RM | 687.177 | 989.878 | 0,0305355 |
| CTB-175P5.4 | Known_lncRNA | D04 | DO4-RM | 0 | 194.789 | 0,0305355 |
| CYP24A1 | ProteinCoding | D04 | DO4-RM | 0 | 0,7391 | 0,0305355 |
| RP11-143A12.3 | Known_lncRNA | D04 | DO4-RM | 0,936388 | 0 | 0,0305355 |
| HMGCLL1 | ProteinCoding | D04 | DO4-RM | 0 | 106.845 | 0,0305355 |
| RASSF9 | ProteinCoding | D04 | DO4-RM | 0 | 269.335 | 0,0352149 |
| ANPEP | ProteinCoding | D04 | DO4-RM | 155.946 | 239.698 | 0,0352149 |
| SERPINE2 | Known_lncRNA | D04 | DO4-RM | 863.805 | 162.037 | 0,0352149 |
| LINC00313 | Known_lncRNA | D04 | DO4-RM | 0 | 0,76027 | 0,0352149 |
| **SPOCK1** | ProteinCoding | D04 | DO4-RM | 419.568 | 635.444 | 0,0352149 |
| CLCN5 | ProteinCoding | D04 | DO4-RM | 111.538 | 0,604634 | 0,0352149 |
| TCONS_00001649_lncRNA | Novel lncRNA | D04 | DO4-RM | 292.481 | 0 | 0,0381142 |
| TCONS_00005512_lncRNA | Novel lncRNA | D04 | DO4-RM | 155.476 | 0 | 0,0381142 |
| NES | ProteinCoding | D04 | DO4-RM | 110.509 | 125.669 | 0,0381142 |
| TCONS_00018568_lncRNA | Novel lncRNA | D04 | DO4-RM | 15.717 | 0 | 0,0381142 |
| ATP2B1 | ProteinCoding | D04 | DO4-RM | 253.375 | 194.969 | 0,0381142 |
| FAM216B | ProteinCoding | D04 | DO4-RM | 0 | 0,500939 | 0,0381142 |
| GAS7 | ProteinCoding | D04 | DO4-RM | 647.961 | 365.431 | 0,0381142 |
| TIMP3 | ProteinCoding | D04 | DO4-RM | 317,82 | 465.171 | 0,0381142 |
| FSTL1 | ProteinCoding | D04 | DO4-RM | 771.275 | 84.619 | 0,0381142 |
| IL7R | ProteinCoding | D04 | DO4-RM | 60.622 | 611.579 | 0,0381142 |
| GPR115 | ProteinCoding | D04 | DO4-RM | 0 | 0,448073 | 0,0381142 |
| SDC2 | ProteinCoding | D04 | DO4-RM | 0,520433 | 703.705 | 0,0381142 |
| TCONS_00015814_lncRNA | Novel lncRNA | D04 | DO4-RM | 414.061 | 0,284038 | 0,0420395 |
| MFAP5 | ProteinCoding | D04 | DO4-RM | 0 | 0,567161 | 0,0420395 |
| C3 | ProteinCoding | D04 | DO4-RM | 0 | 0,389969 | 0,0420395 |
| TRIM2 | ProteinCoding | D04 | DO4-RM | 603.756 | 587.077 | 0,0420395 |
| RHOBTB3 | ProteinCoding | D04 | DO4-RM | 121.999 | 193.982 | 0,0420395 |
| MMP8 | ProteinCoding | D04 | DO4-RM | 596.915 | 0,566673 | 0,0444516 |
| KRT81 | ProteinCoding | D04 | DO4-RM | 0 | 0,680577 | 0,0444516 |
| FBN1 | ProteinCoding | D04 | DO4-RM | 0,138262 | 109.411 | 0,0444516 |
| B3GNT3 | ProteinCoding | D04 | DO4-RM | 0 | 0,500813 | 0,0444516 |
| CD70 | ProteinCoding | D04 | DO4-RM | 0 | 17.605 | 0,0444516 |
| ADRA1D | ProteinCoding | D04 | DO4-RM | 0 | 0,511755 | 0,0444516 |
| PREX1 | ProteinCoding | D04 | DO4-RM | 195.315 | 108.797 | 0,0444516 |
| C4ORF26 | ProteinCoding | D04 | DO4-RM | 0 | 0,403591 | 0,0444516 |
| RP11-44F21.5 | Known_lncRNA | D04 | DO4-RM | 0 | 171.455 | 0,0444516 |
| VOPP1 | ProteinCoding | D04 | DO4-RM | 0 | 0,424625 | 0,0444516 |
| NPBWR1 | ProteinCoding | D04 | DO4-RM | 0 | 237.368 | 0,0444516 |
| TCONS_00003841_lncRNA | Novel lncRNA | D04 | DO4-RM | 14.271 | 0 | 0,0461148 |
| S100A4 | ProteinCoding | D04 | DO4-RM | 276.317 | 1967 | 0,0461148 |
| PRSS23 | ProteinCoding | D04 | DO4-RM | 313.548 | 220.935 | 0,0461148 |
| C11ORF21 | Known_lncRNA | D04 | DO4-RM | 0 | 0,654347 | 0,0461148 |
| CMKLR1 | ProteinCoding | D04 | DO4-RM | 0 | 0,516854 | 0,0461148 |
| MSI1 | ProteinCoding | D04 | DO4-RM | 0,435426 | 0 | 0,0461148 |
| LINC00669 | Known_lncRNA | D04 | DO4-RM | 0,00204713 | 160.473 | 0,0461148 |
| ITGA6 | ProteinCoding | D04 | DO4-RM | 183.569 | 174.871 | 0,0461148 |
| FAM172BP | Known_lncRNA | D04 | DO4-RM | 0 | 0,663907 | 0,0461148 |
| HLA-DRB1 | ProteinCoding | D04 | DO4-RM | 800.595 | 231.528 | 0,0461148 |
| FBXO32 | ProteinCoding | D04 | DO4-RM | 183.857 | 108.265 | 0,0461148 |
| COL5A1 | ProteinCoding | D04 | DO4-RM | 0,527756 | 628.458 | 0,0461148 |
| LOC494127 | Known_lncRNA | D04 | DO4-RM | 159.671 | 0 | 0,0461148 |
| TNFRSF25 | ProteinCoding | D04 | DO4-RM | 0,225435 | 379.476 | 0,0482638 |
| TCONS_00011986_lncRNA | Novel lncRNA | D04 | DO4-RM | 0 | 0,997551 | 0,0482638 |
| RPTN | ProteinCoding | D04 | DO4-RM | 0 | 0,422861 | 0,0482638 |
| RP13-631K18.5 | Known_lncRNA | D04 | DO4-RM | 0 | 0,509206 | 0,0482638 |
| CST7 | ProteinCoding | D04 | DO4-RM | 0 | 194.029 | 0,0482638 |
| CTD-2187J20.1 | Known_lncRNA | D04 | DO4-RM | 0 | 0,511247 | 0,0482638 |
| GCNT4 | ProteinCoding | D04 | DO4-RM | 0 | 0,440211 | 0,0482638 |
| RBM24 | ProteinCoding | D04 | DO4-RM | 0 | 139.947 | 0,0482638 |
| RP11-31F19.1 | Known_lncRNA | D04 | DO4-RM | 513.631 | 483.272 | 0,0482638 |
| MOSPD2 | ProteinCoding | D04 | DO4-RM | 439.815 | 431.404 | 0,0482638 |

*For novel lncRNA and TUCP identified in more samples or by more programs only one transcript name was kept. In red transcript found significant in all samples comparisons.*

***Supplementary Table 2:*** *Genes differentially expressed in MM415-RM compared to MM415. after filtering for genes differently expressed in MM415-A*

| **Names of Transcripts** | **Category** | **sample_1** | **sample_2** | **value_1** | **value_2** | **q_value** |
| --- | --- | --- | --- | --- | --- | --- |
| CDA | ProteinCoding | MM415 | MM415-RM | 0 | 105.241 | 0,0106274 |
| TCONS_00001847_lncRNA | Novel lncRNA | MM415 | MM415-RM | 0 | 557.258 | 0,0106274 |
| TXNIP | ProteinCoding | MM415 | MM415-RM | 367.404 | 186.972 | 0,0106274 |
| TCONS_00006675_lncRNA | Novel lncRNA | MM415 | MM415-RM | 0 | 127.206 | 0,0106274 |
| TCONS_00008022_lncRNA | Novel lncRNA | MM415 | MM415-RM | 0,128929 | 364.929 | 0,0106274 |
| TCONS_00008109_lncRNA | Novel lncRNA | MM415 | MM415-RM | 0,126097 | 246.525 | 0,0106274 |
| TCONS_00011393_lncRNA | Novel lncRNA | MM415 | MM415-RM | 0 | 0,511462 | 0,0106274 |
| TCONS_00011582_lncRNA | Novel lncRNA | MM415 | MM415-RM | 471.026 | 0 | 0,0106274 |
| PDZK1IP1 | ProteinCoding | MM415 | MM415-RM | 0 | 785.966 | 0,0106274 |
| TCONS_00012966_TUCP_Pfam | Novel lncRNA | MM415 | MM415-RM | 0 | 266.675 | 0,0106274 |
| KANK4 | ProteinCoding | MM415 | MM415-RM | 0 | 133.226 | 0,0106274 |
| TCONS_00013834_lncRNA | Novel lncRNA | MM415 | MM415-RM | 0,048692 | 351.241 | 0,0106274 |
| TCONS_00015269_lncRNA | Novel lncRNA | MM415 | MM415-RM | 0,21723 | 838.432 | 0,0106274 |
| TCONS_00016919_lncRNA | Novel lncRNA | MM415 | MM415-RM | 0 | 792.069 | 0,0106274 |
| HHIPL2 | ProteinCoding | MM415 | MM415-RM | 0 | 237.555 | 0,0106274 |
| TCONS_00017166_TUCP_Pfam | Novel lncRNA | MM415 | MM415-RM | 719.224 | 142.538 | 0,0106274 |
| APBB1IP | ProteinCoding | MM415 | MM415-RM | 0 | 424.378 | 0,0106274 |
| **MIRAT** | Novel lncRNA | MM415 | MM415-RM | 0,0983982 | 692.818 | 0,0106274 |
| MAP3K8 | ProteinCoding | MM415 | MM415-RM | 0 | 608.012 | 0,0106274 |
| TCONS_00019025_TUCP_Pfam | TUCP | MM415 | MM415-RM | 0 | 733.222 | 0,0106274 |
| TCONS_00018633_lncRNA | Novel lncRNA | MM415 | MM415-RM | 485.983 | 0,570335 | 0,0106274 |
| MKX | ProteinCoding | MM415 | MM415-RM | 0 | 344.872 | 0,0106274 |
| ARMC4 | ProteinCoding | MM415 | MM415-RM | 0 | 188.458 | 0,0106274 |
| ARHGAP22 | ProteinCoding | MM415 | MM415-RM | 0 | 147.355 | 0,0106274 |
| GRAMD1B | ProteinCoding | MM415 | MM415-RM | 0 | 111.789 | 0,0106274 |
| SLC38A1 | ProteinCoding | MM415 | MM415-RM | 0,850512 | 289.642 | 0,0106274 |
| KRT5 | ProteinCoding | MM415 | MM415-RM | 0 | 568.569 | 0,0106274 |
| TMEM119 | ProteinCoding | MM415 | MM415-RM | 0 | 457.773 | 0,0106274 |
| LINC00643 | Known_lncRNA | MM415 | MM415-RM | 0 | 188.049 | 0,0106274 |
| KIF26A | ProteinCoding | MM415 | MM415-RM | 0 | 111.716 | 0,0106274 |
| DPF3 | ProteinCoding | MM415 | MM415-RM | 0 | 130.028 | 0,0106274 |
| FBN1 | ProteinCoding | MM415 | MM415-RM | 128.027 | 935.241 | 0,0106274 |
| MMP2 | ProteinCoding | MM415 | MM415-RM | 660.099 | 222.763 | 0,0106274 |
| FOXL1 | ProteinCoding | MM415 | MM415-RM | 0 | 0,890399 | 0,0106274 |
| HS3ST3A1 | ProteinCoding | MM415 | MM415-RM | 0 | 142.219 | 0,0106274 |
| KRT17 | ProteinCoding | MM415 | MM415-RM | 0 | 675.738 | 0,0106274 |
| ADCYAP1 | ProteinCoding | MM415 | MM415-RM | 0 | 0,809011 | 0,0106274 |
| PALM | ProteinCoding | MM415 | MM415-RM | 0 | 714.707 | 0,0106274 |
| EMR1 | ProteinCoding | MM415 | MM415-RM | 0 | 29.373 | 0,0106274 |
| IFI30 | ProteinCoding | MM415 | MM415-RM | 806.998 | 210.199 | 0,0106274 |
| PSG5 | ProteinCoding | MM415 | MM415-RM | 0 | 264.891 | 0,0106274 |
| IL1RN | ProteinCoding | MM415 | MM415-RM | 0 | 133.603 | 0,0106274 |
| LYPD6B | ProteinCoding | MM415 | MM415-RM | 0 | 968.561 | 0,0106274 |
| AC012507.3 | Known_lncRNA | MM415 | MM415-RM | 0,447055 | 0 | 0,0106274 |
| OSR1 | ProteinCoding | MM415 | MM415-RM | 0 | 357.882 | 0,0106274 |
| PID1 | ProteinCoding | MM415 | MM415-RM | 0 | 144.856 | 0,0106274 |
| GPR55 | ProteinCoding | MM415 | MM415-RM | 125.215 | 0 | 0,0106274 |
| TGM3 | ProteinCoding | MM415 | MM415-RM | 0 | 138.649 | 0,0106274 |
| BMP2 | ProteinCoding | MM415 | MM415-RM | 0 | 341.511 | 0,0106274 |
| LINC00320 | Known_lncRNA | MM415 | MM415-RM | 0 | 0,485715 | 0,0106274 |
| S100B | ProteinCoding | MM415 | MM415-RM | 527.577 | 386.868 | 0,0106274 |
| KIAA1644 | ProteinCoding | MM415 | MM415-RM | 0 | 174.273 | 0,0106274 |
| AGTR1 | ProteinCoding | MM415 | MM415-RM | 0 | 225.822 | 0,0106274 |
| TP63 | ProteinCoding | MM415 | MM415-RM | 0 | 109.814 | 0,0106274 |
| NPNT | ProteinCoding | MM415 | MM415-RM | 0 | 451.212 | 0,0106274 |
| TLL1 | ProteinCoding | MM415 | MM415-RM | 0 | 186.995 | 0,0106274 |
| SFRP2 | ProteinCoding | MM415 | MM415-RM | 0 | 880.399 | 0,0106274 |
| GCNT4 | ProteinCoding | MM415 | MM415-RM | 0 | 834.653 | 0,0106274 |
| TPBG | ProteinCoding | MM415 | MM415-RM | 0 | 619.852 | 0,0106274 |
| UNC5CL | ProteinCoding | MM415 | MM415-RM | 26.502 | 0 | 0,0106274 |
| COL21A1 | ProteinCoding | MM415 | MM415-RM | 0 | 237.621 | 0,0106274 |
| COL12A1 | ProteinCoding | MM415 | MM415-RM | 236.266 | 256.383 | 0,0106274 |
| ROS1 | ProteinCoding | MM415 | MM415-RM | 0 | 0,752349 | 0,0106274 |
| BMPER | ProteinCoding | MM415 | MM415-RM | 0 | 502.727 | 0,0106274 |
| ELN | ProteinCoding | MM415 | MM415-RM | 0 | 280.661 | 0,0106274 |
| KCND2 | ProteinCoding | MM415 | MM415-RM | 0 | 126.533 | 0,0106274 |
| ASNS | ProteinCoding | MM415 | MM415-RM | 307.254 | 12.042 | 0,0106274 |
| PPP1R3A | ProteinCoding | MM415 | MM415-RM | 0 | 383.885 | 0,0106274 |
| TSPAN12 | ProteinCoding | MM415 | MM415-RM | 0 | 791.704 | 0,0106274 |
| TMEM176B | ProteinCoding | MM415 | MM415-RM | 0 | 132.425 | 0,0106274 |
| SULF1 | ProteinCoding | MM415 | MM415-RM | 0 | 280.019 | 0,0106274 |
| COLEC10 | ProteinCoding | MM415 | MM415-RM | 0 | 791.655 | 0,0106274 |
| MSR1 | ProteinCoding | MM415 | MM415-RM | 0 | 128.278 | 0,0106274 |
| RUNX1T1 | ProteinCoding | MM415 | MM415-RM | 0 | 313.314 | 0,0106274 |
| CNTNAP3B | ProteinCoding | MM415 | MM415-RM | 0,851088 | 0 | 0,0106274 |
| KLHL4 | ProteinCoding | MM415 | MM415-RM | 0 | 282.263 | 0,0106274 |
| COL4A5 | ProteinCoding | MM415 | MM415-RM | 0 | 362.729 | 0,0106274 |
| GRIA3 | ProteinCoding | MM415 | MM415-RM | 0 | 183.544 | 0,0106274 |
| CLIC2 | ProteinCoding | MM415 | MM415-RM | 0 | 129.929 | 0,0106274 |
| ST6GALNAC5 | ProteinCoding | MM415 | MM415-RM | 0 | 16.454 | 0,0181552 |
| TCONS_00012141_TUCP_Pfam | Novel lncRNA | MM415 | MM415-RM | 0 | 0,483443 | 0,0181552 |
| MUC1 | ProteinCoding | MM415 | MM415-RM | 0,107324 | 158.477 | 0,0181552 |
| ADAMTS14 | ProteinCoding | MM415 | MM415-RM | 0 | 0,568811 | 0,0181552 |
| STYK1 | ProteinCoding | MM415 | MM415-RM | 0 | 106.911 | 0,0181552 |
| LUM | ProteinCoding | MM415 | MM415-RM | 717.372 | 527.858 | 0,0181552 |
| KRTAP1-5 | ProteinCoding | MM415 | MM415-RM | 0 | 33.806 | 0,0181552 |
| APOC4-APOC2 | Known_lncRNA | MM415 | MM415-RM | 130.066 | 0 | 0,0181552 |
| GALNT13 | ProteinCoding | MM415 | MM415-RM | 0 | 0,519268 | 0,0181552 |
| RP11-38P22.2 | Known_lncRNA | MM415 | MM415-RM | 100.725 | 0 | 0,0181552 |
| LTF | ProteinCoding | MM415 | MM415-RM | 0 | 0,778829 | 0,0181552 |
| FOXQ1 | ProteinCoding | MM415 | MM415-RM | 0 | 107.858 | 0,0181552 |
| EDN1 | ProteinCoding | MM415 | MM415-RM | 0 | 152.296 | 0,0181552 |
| FOXE1 | ProteinCoding | MM415 | MM415-RM | 0 | 169.683 | 0,0181552 |
| IFI6 | ProteinCoding | MM415 | MM415-RM | 600.079 | 884,84 | 0,0239849 |
| TCONS_00019734_lncRNA | Novel lncRNA | MM415 | MM415-RM | 0 | 134.155 | 0,0239849 |
| STON2 | ProteinCoding | MM415 | MM415-RM | 0,486247 | 36.012 | 0,0239849 |
| RLBP1 | ProteinCoding | MM415 | MM415-RM | 375.782 | 0 | 0,0239849 |
| FOXF1 | ProteinCoding | MM415 | MM415-RM | 0 | 129.754 | 0,0239849 |
| MARCH4 | ProteinCoding | MM415 | MM415-RM | 0 | 0,579728 | 0,0239849 |
| PTPRN | ProteinCoding | MM415 | MM415-RM | 0 | 0,715217 | 0,0239849 |
| PAK7 | ProteinCoding | MM415 | MM415-RM | 0 | 0,466739 | 0,0239849 |
| SCUBE1 | ProteinCoding | MM415 | MM415-RM | 0 | 0,917117 | 0,0239849 |
| ASB5 | ProteinCoding | MM415 | MM415-RM | 0 | 137.473 | 0,0239849 |
| FAM83B | ProteinCoding | MM415 | MM415-RM | 0 | 0,741915 | 0,0239849 |
| IGFBP3 | ProteinCoding | MM415 | MM415-RM | 44.887 | 242.434 | 0,0239849 |
| PNMA2 | ProteinCoding | MM415 | MM415-RM | 0 | 0,769358 | 0,0239849 |
| TCONS_00011531_lncRNA | Novel lncRNA | MM415 | MM415-RM | 0,642917 | 0 | 0,0276651 |
| HTRA1 | ProteinCoding | MM415 | MM415-RM | 160.344 | 285.922 | 0,0276651 |
| TTC9 | ProteinCoding | MM415 | MM415-RM | 0 | 109.782 | 0,0276651 |
| KRT34 | ProteinCoding | MM415 | MM415-RM | 0 | 128.848 | 0,0276651 |
| ZNF442 | ProteinCoding | MM415 | MM415-RM | 0 | 0,670128 | 0,0276651 |
| TMEM37 | ProteinCoding | MM415 | MM415-RM | 0 | 131.062 | 0,0276651 |
| RP5-839B4.8 | Known_lncRNA | MM415 | MM415-RM | 0 | 125.652 | 0,0276651 |
| CHL1 | ProteinCoding | MM415 | MM415-RM | 48,11 | 0,956168 | 0,0276651 |
| AC144521.1 | Known_lncRNA | MM415 | MM415-RM | 0,00363559 | 0,454769 | 0,0276651 |
| RP11-1299A16.3 | Known_lncRNA | MM415 | MM415-RM | 0 | 0,807743 | 0,0276651 |
| RASGEF1B | ProteinCoding | MM415 | MM415-RM | 0 | 134.096 | 0,0276651 |
| **SPOCK1** | ProteinCoding | MM415 | MM415-RM | 0,728217 | 125.962 | 0,0276651 |
| SOD2 | ProteinCoding | MM415 | MM415-RM | 412.565 | 644,52 | 0,0276651 |
| NOV | ProteinCoding | MM415 | MM415-RM | 541.508 | 173.139 | 0,0276651 |
| ENPP2 | Known_lncRNA | MM415 | MM415-RM | 260.841 | 120.439 | 0,0276651 |
| ZC4H2 | Known_lncRNA | MM415 | MM415-RM | 0 | 0,564344 | 0,0276651 |
| TCONS_00000730_TUCP_Pfam | Novel lncRNA | MM415 | MM415-RM | 300.121 | 0 | 0,0332615 |
| PHGDH | ProteinCoding | MM415 | MM415-RM | 460.545 | 212.287 | 0,0332615 |
| TCONS_00019602_TUCP_Pfam | Novel lncRNA | MM415 | MM415-RM | 690.127 | 108.776 | 0,0332615 |
| IFI27 | ProteinCoding | MM415 | MM415-RM | 379.558 | 507,36 | 0,0332615 |
| CYP2J2 | ProteinCoding | MM415 | MM415-RM | 0 | 0,981876 | 0,037083 |
| TCONS_00015278_lncRNA | Novel lncRNA | MM415 | MM415-RM | 0 | 132.513 | 0,037083 |
| TCONS_00018149_lncRNA | Novel lncRNA | MM415 | MM415-RM | 0 | 291.266 | 0,037083 |
| TCONS_00019733_TUCP_Pfam | TUCP | MM415 | MM415-RM | 0 | 136.277 | 0,037083 |
| SMCO4 | ProteinCoding | MM415 | MM415-RM | 0 | 211.436 | 0,037083 |
| FPR1 | ProteinCoding | MM415 | MM415-RM | 0 | 159.025 | 0,037083 |
| KCTD8 | ProteinCoding | MM415 | MM415-RM | 0 | 0,888103 | 0,037083 |
| DPY19L2P1 | Known_lncRNA | MM415 | MM415-RM | 0 | 0,704218 | 0,037083 |
| HS3ST2 | ProteinCoding | MM415 | MM415-RM | 0 | 118.501 | 0,0417818 |
| SAA1 | ProteinCoding | MM415 | MM415-RM | 0 | 347.922 | 0,0417819 |
| POTEG | Known_lncRNA | MM415 | MM415-RM | 0 | 110.106 | 0,0417819 |
| TMEM92 | ProteinCoding | MM415 | MM415-RM | 0 | 10.498 | 0,0417819 |
| FPR2 | ProteinCoding | MM415 | MM415-RM | 0 | 313.586 | 0,0417819 |
| HHIPL1 | ProteinCoding | MM415 | MM415-RM | 0 | 118.115 | 0,045566 |
| NOG | ProteinCoding | MM415 | MM415-RM | 0 | 0,817594 | 0,045566 |
| DSEL | ProteinCoding | MM415 | MM415-RM | 0,323893 | 199.436 | 0,045566 |
| GMNC | ProteinCoding | MM415 | MM415-RM | 0 | 110.173 | 0,045566 |
| CPE | ProteinCoding | MM415 | MM415-RM | 0 | 104.475 | 0,045566 |
| VGF | ProteinCoding | MM415 | MM415-RM | 128.903 | 130.245 | 0,045566 |
| APLN | ProteinCoding | MM415 | MM415-RM | 0 | 0,460555 | 0,045566 |
| TCONS_00005450_TUCP_Pfam | Novel lncRNA | MM415 | MM415-RM | 0,154739 | 10.486 | 0,0487146 |
| TCONS_00017521_lncRNA | Novel lncRNA | MM415 | MM415-RM | 0 | 10.216 | 0,0487146 |
| C1S | ProteinCoding | MM415 | MM415-RM | 281.147 | 193.624 | 0,0487146 |
| MCF2L | ProteinCoding | MM415 | MM415-RM | 0,387759 | 304.161 | 0,0487146 |
| MYCT1 | ProteinCoding | MM415 | MM415-RM | 0 | 0,432008 | 0,0487146 |
| TMSB4X | ProteinCoding | MM415 | MM415-RM | 85.048 | 994.815 | 0,0487146 |
| TMEM255A | ProteinCoding | MM415 | MM415-RM | 215.134 | 329.888 | 0,0487146 |

*For novel lncRNA and TUCP identified in more samples or by more programs only one transcript name was kept. In red transcript found significant in all samples comparisons.*

***Supplementary Table 3:*** *Genes differentially expressed in AV4-RM compared to AV4. after filtering for genes differently expressed in AV5-ChrExp.*

| **Names of Transcripts** | **Category** | **sample_1** | **sample_2** | **value_1** | **value_2** | **q_value** |
| --- | --- | --- | --- | --- | --- | --- |
| TCONS_00000514_TUCP_Pfam | Novel lncRNA | AV4 | AV4-RM | 0 | 235.729 | 0,0124271 |
| TCONS_00001834_TUCP_Pfam | Novel lncRNA | AV4 | AV4-RM | 0,850515 | 249.305 | 0,0124271 |
| TCONS_00002273_lncRNA | Novel lncRNA | AV4 | AV4-RM | 0,00745219 | 0,740627 | 0,0124271 |
| PTGFR | ProteinCoding | AV4 | AV4-RM | 0,317405 | 183.459 | 0,0124271 |
| TCONS_00005450_TUCP_Pfam | Novel lncRNA | AV4 | AV4-RM | 0,0495239 | 147.601 | 0,0124271 |
| CFH | ProteinCoding | AV4 | AV4-RM | 670.164 | 700.575 | 0,0124271 |
| FMN2 | ProteinCoding | AV4 | AV4-RM | 605.976 | 840.712 | 0,0124271 |
| TCONS_00011393_lncRNA | Novel lncRNA | AV4 | AV4-RM | 0 | 0,401055 | 0,0124271 |
| TCONS_00011689_lncRNA | Novel lncRNA | AV4 | AV4-RM | 227.549 | 0 | 0,0124271 |
| TCONS_00014413_lncRNA | Novel lncRNA | AV4 | AV4-RM | 0,0100279 | 183.297 | 0,0124271 |
| TCONS_00017166_TUCP_Pfam | Novel lncRNA | AV4 | AV4-RM | 578.808 | 953.654 | 0,0124271 |
| RGS7 | ProteinCoding | AV4 | AV4-RM | 0 | 0,950186 | 0,0124271 |
| **MIRAT** | Novel lncRNA | AV4 | AV4-RM | 0,00793972 | 570.687 | 0,0124271 |
| TCONS_00018871_lncRNA | Novel lncRNA | AV4 | AV4-RM | 435.438 | 199.749 | 0,0124271 |
| PLAU | ProteinCoding | AV4 | AV4-RM | 122.408 | 172.325 | 0,0124271 |
| CXCL12 | ProteinCoding | AV4 | AV4-RM | 0 | 135.361 | 0,0124271 |
| HPSE2 | ProteinCoding | AV4 | AV4-RM | 0,390154 | 0 | 0,0124271 |
| TYR | ProteinCoding | AV4 | AV4-RM | 157.333 | 681.224 | 0,0124271 |
| MMP8 | ProteinCoding | AV4 | AV4-RM | 153.936 | 107.984 | 0,0124271 |
| MYBPC1 | ProteinCoding | AV4 | AV4-RM | 0,990451 | 0 | 0,0124271 |
| CD163L1 | ProteinCoding | AV4 | AV4-RM | 112.078 | 221.191 | 0,0124271 |
| LUM | ProteinCoding | AV4 | AV4-RM | 216.654 | 319.916 | 0,0124271 |
| TNFRSF19 | ProteinCoding | AV4 | AV4-RM | 125.811 | 146.141 | 0,0124271 |
| SHISA2 | ProteinCoding | AV4 | AV4-RM | 146.192 | 466.064 | 0,0124271 |
| POSTN | ProteinCoding | AV4 | AV4-RM | 129.641 | 175.504 | 0,0124271 |
| TRPC4 | ProteinCoding | AV4 | AV4-RM | 0 | 20.536 | 0,0124271 |
| DCT | ProteinCoding | AV4 | AV4-RM | 221,79 | 645.768 | 0,0124271 |
| SERPINA3 | ProteinCoding | AV4 | AV4-RM | 280,4 | 547.118 | 0,0124271 |
| GREM1 | ProteinCoding | AV4 | AV4-RM | 0,446879 | 412.101 | 0,0124271 |
| THBS1 | ProteinCoding | AV4 | AV4-RM | 231.403 | 465.419 | 0,0124271 |
| CRISPLD2 | ProteinCoding | AV4 | AV4-RM | 326.884 | 336.028 | 0,0124271 |
| GAS7 | ProteinCoding | AV4 | AV4-RM | 52.799 | 779.748 | 0,0124271 |
| RP11-527H14.2 | Known_lncRNA | AV4 | AV4-RM | 0,489216 | 738.969 | 0,0124271 |
| RAB27B | ProteinCoding | AV4 | AV4-RM | 586.613 | 119.344 | 0,0124271 |
| COLEC12 | ProteinCoding | AV4 | AV4-RM | 0,739485 | 265.945 | 0,0124271 |
| RP11-805F19.1 | Known_lncRNA | AV4 | AV4-RM | 21.564 | 0 | 0,0124271 |
| SERPINB4 | ProteinCoding | AV4 | AV4-RM | 144.189 | 789.396 | 0,0124271 |
| CDH19 | Known_lncRNA | AV4 | AV4-RM | 171.086 | 954.126 | 0,0124271 |
| PSG11 | ProteinCoding | AV4 | AV4-RM | 0 | 31.958 | 0,0124271 |
| PSG2 | ProteinCoding | AV4 | AV4-RM | 0 | 842.584 | 0,0124271 |
| COL3A1 | ProteinCoding | AV4 | AV4-RM | 22.237 | 719.766 | 0,0124271 |
| AOX1 | ProteinCoding | AV4 | AV4-RM | 0,490642 | 326.581 | 0,0124271 |
| UGT1A8 | ProteinCoding | AV4 | AV4-RM | 0 | 55.716 | 0,0124271 |
| OSR1 | ProteinCoding | AV4 | AV4-RM | 0 | 542.595 | 0,0124271 |
| RP1-156L9.1 | Known_lncRNA | AV4 | AV4-RM | 0,562866 | 0 | 0,0124271 |
| COL6A3 | ProteinCoding | AV4 | AV4-RM | 50.723 | 134.955 | 0,0124271 |
| SPTLC3 | ProteinCoding | AV4 | AV4-RM | 0 | 222.385 | 0,0124271 |
| CPXM1 | ProteinCoding | AV4 | AV4-RM | 0,870235 | 0 | 0,0124271 |
| JPH2 | ProteinCoding | AV4 | AV4-RM | 0 | 454.683 | 0,0124271 |
| PVRL3 | ProteinCoding | AV4 | AV4-RM | 329.291 | 678.557 | 0,0124271 |
| TF | ProteinCoding | AV4 | AV4-RM | 126,06 | 152.412 | 0,0124271 |
| MME | ProteinCoding | AV4 | AV4-RM | 212.933 | 318.032 | 0,0124271 |
| WNT5A | ProteinCoding | AV4 | AV4-RM | 356.902 | 746.661 | 0,0124271 |
| VGLL3 | ProteinCoding | AV4 | AV4-RM | 0 | 426.862 | 0,0124271 |
| FSTL1 | ProteinCoding | AV4 | AV4-RM | 230.213 | 175.239 | 0,0124271 |
| CPZ | ProteinCoding | AV4 | AV4-RM | 0 | 161.716 | 0,0124271 |
| CXCL5 | ProteinCoding | AV4 | AV4-RM | 682.225 | 189.613 | 0,0124271 |
| ANTXR2 | ProteinCoding | AV4 | AV4-RM | 492.845 | 402.288 | 0,0124271 |
| CDH18 | ProteinCoding | AV4 | AV4-RM | 0 | 0,643517 | 0,0124271 |
| ADAMTS12 | ProteinCoding | AV4 | AV4-RM | 0 | 350.924 | 0,0124271 |
| STC2 | ProteinCoding | AV4 | AV4-RM | 808.878 | 703.246 | 0,0124271 |
| ROS1 | ProteinCoding | AV4 | AV4-RM | 0 | 0,353312 | 0,0124271 |
| PTPRK | ProteinCoding | AV4 | AV4-RM | 353.652 | 415.286 | 0,0124271 |
| COL1A2 | ProteinCoding | AV4 | AV4-RM | 449.179 | 114.725 | 0,0124271 |
| TAC1 | ProteinCoding | AV4 | AV4-RM | 193.823 | 0 | 0,0124271 |
| NM_001165413 | ProteinCoding | AV4 | AV4-RM | 892.805 | 926.582 | 0,0124271 |
| CPED1 | ProteinCoding | AV4 | AV4-RM | 0,971281 | 310.199 | 0,0124271 |
| HAS2 | ProteinCoding | AV4 | AV4-RM | 392.816 | 62.132 | 0,0124271 |
| COL15A1 | ProteinCoding | AV4 | AV4-RM | 370.443 | 490.613 | 0,0124271 |
| PAPPA | ProteinCoding | AV4 | AV4-RM | 0,254717 | 634.282 | 0,0124271 |
| COL5A1 | ProteinCoding | AV4 | AV4-RM | 118.317 | 194.735 | 0,0124271 |
| CDR1 | ProteinCoding | AV4 | AV4-RM | 0 | 286.112 | 0,0124271 |
| TGFB2 | ProteinCoding | AV4 | AV4-RM | 10.704 | 218.373 | 0,0218232 |
| RAB3B | ProteinCoding | AV4 | AV4-RM | 0,405703 | 84.543 | 0,0218232 |
| ITIH5 | ProteinCoding | AV4 | AV4-RM | 316.782 | 155.421 | 0,0218232 |
| RP11-720L2.4 | Known_lncRNA | AV4 | AV4-RM | 0 | 124.982 | 0,0218232 |
| MIA | ProteinCoding | AV4 | AV4-RM | 245.012 | 309.052 | 0,0218232 |
| C3 | ProteinCoding | AV4 | AV4-RM | 110.266 | 746.182 | 0,0218232 |
| DPP4 | ProteinCoding | AV4 | AV4-RM | 0,619097 | 260.155 | 0,0218232 |
| COL6A2 | ProteinCoding | AV4 | AV4-RM | 259.716 | 162.365 | 0,0218232 |
| CCDC80 | ProteinCoding | AV4 | AV4-RM | 223.871 | 272.311 | 0,0218232 |
| ENPP2 | Known_lncRNA | AV4 | AV4-RM | 165.866 | 223.659 | 0,0218232 |
| KITLG | ProteinCoding | AV4 | AV4-RM | 194.443 | 199.357 | 0,0301601 |
| COL1A1 | ProteinCoding | AV4 | AV4-RM | 0,525304 | 245.058 | 0,0301601 |
| CTD-2541J13.1 | Known_lncRNA | AV4 | AV4-RM | 0 | 0,431308 | 0,0301601 |
| COL6A1 | ProteinCoding | AV4 | AV4-RM | 158.287 | 106.027 | 0,0301601 |
| STK32A | ProteinCoding | AV4 | AV4-RM | 157.873 | 0 | 0,0301601 |
| IL6 | ProteinCoding | AV4 | AV4-RM | 124.841 | 687.418 | 0,0301601 |
| EGFR | ProteinCoding | AV4 | AV4-RM | 114.159 | 229.501 | 0,0301601 |
| RGS1 | ProteinCoding | AV4 | AV4-RM | 36.895 | 179.849 | 0,0368969 |
| F3 | ProteinCoding | AV4 | AV4-RM | 2.517 | 593.093 | 0,0368969 |
| TNFSF4 | ProteinCoding | AV4 | AV4-RM | 15.351 | 264.734 | 0,0368969 |
| SERPINB7 | ProteinCoding | AV4 | AV4-RM | 126.918 | 344.438 | 0,0368969 |
| CRIM1 | ProteinCoding | AV4 | AV4-RM | 561.962 | 464.388 | 0,0368969 |
| **SPOCK1** | ProteinCoding | AV4 | AV4-RM | 0,656266 | 234.145 | 0,0368969 |
| ADAM19 | ProteinCoding | AV4 | AV4-RM | 240.862 | 280.103 | 0,0368969 |
| PTPRZ1 | ProteinCoding | AV4 | AV4-RM | 628.948 | 10.217 | 0,0368969 |
| VCAM1 | ProteinCoding | AV4 | AV4-RM | 0,981474 | 403.171 | 0,0451894 |
| AC004603.4 | Known_lncRNA | AV4 | AV4-RM | 0 | 0,472696 | 0,0451894 |
| TCONS_00000254_TUCP_Pfam | TUCP | AV4 | AV4-RM | 0 | 128.861 | 0,0492523 |
| MARCKSL1 | Known_lncRNA | AV4 | AV4-RM | 715.794 | 639.227 | 0,0492523 |
| TCONS_00017704_lncRNA | Novel lncRNA | AV4 | AV4-RM | 0 | 132.577 | 0,0492523 |
| CHST11 | ProteinCoding | AV4 | AV4-RM | 40.026 | 703.692 | 0,0492523 |
| RAD21L1 | ProteinCoding | AV4 | AV4-RM | 0 | 100.878 | 0,0492523 |
| CHL1 | ProteinCoding | AV4 | AV4-RM | 509.897 | 0,261099 | 0,0492523 |
| VEGFC | ProteinCoding | AV4 | AV4-RM | 0,946672 | 54.579 | 0,0492523 |
| CSF2 | ProteinCoding | AV4 | AV4-RM | 147.726 | 146.534 | 0,0492523 |
| RP11-1002K11.1 | Known_lncRNA | AV4 | AV4-RM | 115.927 | 246.374 | 0,0492523 |
| ZCCHC5 | ProteinCoding | AV4 | AV4-RM | 0 | 0,662398 | 0,0492523 |

*For novel lncRNA and TUCP identified in more samples or by more programs only one transcript name was kept. In red transcript found significant in all samples comparisons.*

***Supplementary Table 4:*** *Genes differentially expressed in AV1-RM compared to AV1. after filtering for genes differently expressed in AV5-ChrExp.*

| **Names of Transcripts** | **Category** | **sample_1** | **sample_2** | **value_1** | **value_2** | **q_value** |
| --- | --- | --- | --- | --- | --- | --- |
| TCONS_00001777_TUCP_Pfam | Novel lncRNA | AV1 | AV1-RM | 830.314 | 0,106251 | 0,0164535 |
| TCONS_00002273_lncRNA | Novel lncRNA | AV1 | AV1-RM | 0,210345 | 732.632 | 0,0164535 |
| S100A9 | ProteinCoding | AV1 | AV1-RM | 0 | 17.549 | 0,0164535 |
| NHLH1 | ProteinCoding | AV1 | AV1-RM | 0,89372 | 0 | 0,0164535 |
| TCONS_00004888_TUCP_CPAT | Novel lncRNA | AV1 | AV1-RM | 0,51698 | 0,0175125 | 0,0164535 |
| LHX9 | ProteinCoding | AV1 | AV1-RM | 0 | 129.387 | 0,0164535 |
| TCONS_00006970_lncRNA | Novel lncRNA | AV1 | AV1-RM | 0 | 346.186 | 0,0164535 |
| TCONS_00007096_TUCP_Pfam | TUCP | AV1 | AV1-RM | 0 | 159.083 | 0,0164535 |
| TCONS_00013834_lncRNA | Novel lncRNA | AV1 | AV1-RM | 0,46191 | 622.404 | 0,0164535 |
| AIM2 | ProteinCoding | AV1 | AV1-RM | 14.773 | 0 | 0,0164535 |
| TCONS_00014674_lncRNA | Novel lncRNA | AV1 | AV1-RM | 360.125 | 0 | 0,0164535 |
| TCONS_00015269_lncRNA | Novel lncRNA | AV1 | AV1-RM | 0,13662 | 286.621 | 0,0164535 |
| TCONS_00014865_lncRNA | Novel lncRNA | AV1 | AV1-RM | 763.298 | 0 | 0,0164535 |
| TCONS_00015292_lncRNA | Novel lncRNA | AV1 | AV1-RM | 308.437 | 0 | 0,0164535 |
| **MIRAT** | Novel lncRNA | AV1 | AV1-RM | 0,707414 | 170,68 | 0,0164535 |
| TCONS_00018212_lncRNA | Novel lncRNA | AV1 | AV1-RM | 131.768 | 0 | 0,0164535 |
| TCONS_00018633_lncRNA | Novel lncRNA | AV1 | AV1-RM | 0,0337376 | 0,917894 | 0,0164535 |
| TYR | ProteinCoding | AV1 | AV1-RM | 166.845 | 0 | 0,0164535 |
| POSTN | ProteinCoding | AV1 | AV1-RM | 310.447 | 640.507 | 0,0164535 |
| SLITRK1 | ProteinCoding | AV1 | AV1-RM | 0 | 323.806 | 0,0164535 |
| SSTR1 | ProteinCoding | AV1 | AV1-RM | 0 | 633.636 | 0,0164535 |
| CTSG | ProteinCoding | AV1 | AV1-RM | 0 | 323.538 | 0,0164535 |
| RP11-930O11.2 | Known_lncRNA | AV1 | AV1-RM | 0,75219 | 0 | 0,0164535 |
| KRTAP2-3 | ProteinCoding | AV1 | AV1-RM | 0 | 359.297 | 0,0164535 |
| RP11-482H16.1 | Known_lncRNA | AV1 | AV1-RM | 0,074385 | 348.982 | 0,0164535 |
| COL3A1 | ProteinCoding | AV1 | AV1-RM | 172.916 | 206,96 | 0,0164535 |
| AC079779.5 | Known_lncRNA | AV1 | AV1-RM | 135.703 | 0 | 0,0164535 |
| PDE1A | ProteinCoding | AV1 | AV1-RM | 0 | 253.583 | 0,0164535 |
| UBE2E2 | ProteinCoding | AV1 | AV1-RM | 0 | 158.038 | 0,0164535 |
| STAC | ProteinCoding | AV1 | AV1-RM | 0 | 166.343 | 0,0164535 |
| ROBO2 | ProteinCoding | AV1 | AV1-RM | 0 | 158.973 | 0,0164535 |
| ROPN1B | ProteinCoding | AV1 | AV1-RM | 828.754 | 0 | 0,0164535 |
| TF | ProteinCoding | AV1 | AV1-RM | 172.266 | 31.757 | 0,0164535 |
| LINC00635 | Known_lncRNA | AV1 | AV1-RM | 0,643062 | 0,00165592 | 0,0164535 |
| FDCSP | ProteinCoding | AV1 | AV1-RM | 643.467 | 0 | 0,0164535 |
| C4ORF22 | ProteinCoding | AV1 | AV1-RM | 0 | 332.092 | 0,0164535 |
| PCDH10 | ProteinCoding | AV1 | AV1-RM | 0 | 171.741 | 0,0164535 |
| EDNRA | ProteinCoding | AV1 | AV1-RM | 0 | 137.702 | 0,0164535 |
| CXCL3 | ProteinCoding | AV1 | AV1-RM | 249.463 | 0 | 0,0164535 |
| UNC5C | ProteinCoding | AV1 | AV1-RM | 0 | 177.838 | 0,0164535 |
| ASB5 | ProteinCoding | AV1 | AV1-RM | 0 | 126.562 | 0,0164535 |
| CTC-340A15.2 | Known_lncRNA | AV1 | AV1-RM | 0,00216465 | 174.206 | 0,0164535 |
| SPDL1 | ProteinCoding | AV1 | AV1-RM | 314.317 | 120.254 | 0,0164535 |
| RANBP17 | ProteinCoding | AV1 | AV1-RM | 0,663167 | 0 | 0,0164535 |
| CDH18 | ProteinCoding | AV1 | AV1-RM | 0 | 107.204 | 0,0164535 |
| CDH9 | ProteinCoding | AV1 | AV1-RM | 0 | 365.825 | 0,0164535 |
| RIMS1 | ProteinCoding | AV1 | AV1-RM | 0 | 242.421 | 0,0164535 |
| VGLL2 | ProteinCoding | AV1 | AV1-RM | 0 | 552.257 | 0,0164535 |
| COL12A1 | ProteinCoding | AV1 | AV1-RM | 495.968 | 103.835 | 0,0164535 |
| KCND2 | ProteinCoding | AV1 | AV1-RM | 0 | 108.605 | 0,0164535 |
| LMOD2 | ProteinCoding | AV1 | AV1-RM | 111.349 | 0 | 0,0164535 |
| AC009276.4 | Known_lncRNA | AV1 | AV1-RM | 0,66856 | 0 | 0,0164535 |
| SOSTDC1 | ProteinCoding | AV1 | AV1-RM | 525.246 | 0 | 0,0164535 |
| RP11-21C17.1 | Known_lncRNA | AV1 | AV1-RM | 0 | 425.535 | 0,0164535 |
| CYBB | ProteinCoding | AV1 | AV1-RM | 0 | 1.906 | 0,0164535 |
| PLP1 | ProteinCoding | AV1 | AV1-RM | 291.373 | 242.493 | 0,0164535 |
| NXF3 | ProteinCoding | AV1 | AV1-RM | 0 | 168.604 | 0,0164535 |
| TCONS_00000058_lncRNA | Novel lncRNA | AV1 | AV1-RM | 215.359 | 0 | 0,0288569 |
| TCONS_00003856_lncRNA | Novel lncRNA | AV1 | AV1-RM | 122.015 | 0 | 0,0288569 |
| GLUL | ProteinCoding | AV1 | AV1-RM | 195.335 | 862.923 | 0,0288569 |
| NTF3 | ProteinCoding | AV1 | AV1-RM | 0 | 26.399 | 0,0288569 |
| AP1M2 | ProteinCoding | AV1 | AV1-RM | 0 | 130.983 | 0,0288569 |
| EFEMP1 | ProteinCoding | AV1 | AV1-RM | 298.832 | 467.386 | 0,0288569 |
| RP11-48B3.3 | Known_lncRNA | AV1 | AV1-RM | 0,501365 | 0 | 0,0288569 |
| SAT1 | Known_lncRNA | AV1 | AV1-RM | 942.212 | 683.382 | 0,0288569 |
| TCONS_00008109_lncRNA | Novel lncRNA | AV1 | AV1-RM | 0,305104 | 361.924 | 0,0370204 |
| DKK1 | ProteinCoding | AV1 | AV1-RM | 572.182 | 171.948 | 0,0370204 |
| HBB | ProteinCoding | AV1 | AV1-RM | 0 | 165.025 | 0,0370204 |
| RERG | ProteinCoding | AV1 | AV1-RM | 0 | 228.455 | 0,0370204 |
| TMTC1 | ProteinCoding | AV1 | AV1-RM | 871.848 | 133,76 | 0,0370204 |
| NCAM2 | ProteinCoding | AV1 | AV1-RM | 269.044 | 104.162 | 0,0370204 |
| AF241725.6 | Known_lncRNA | AV1 | AV1-RM | 0,166877 | 260.536 | 0,0370204 |
| SEMA3D | ProteinCoding | AV1 | AV1-RM | 0,553672 | 740.918 | 0,0370204 |
| ZDHHC2 | ProteinCoding | AV1 | AV1-RM | 220.923 | 438.987 | 0,0370204 |
| C8ORF34 | ProteinCoding | AV1 | AV1-RM | 0 | 114.201 | 0,0370204 |
| CNKSR2 | ProteinCoding | AV1 | AV1-RM | 0 | 0,809591 | 0,0370204 |
| TCONS_00014413_lncRNA | Novel lncRNA | AV1 | AV1-RM | 0,630032 | 122.102 | 0,0451976 |
| TCONS_00014728_lncRNA | Novel lncRNA | AV1 | AV1-RM | 142.716 | 0 | 0,0451976 |
| SERPINF1 | ProteinCoding | AV1 | AV1-RM | 177.977 | 653.566 | 0,0451976 |
| OXTR | ProteinCoding | AV1 | AV1-RM | 0,578601 | 488.073 | 0,0451976 |
| FSTL1 | ProteinCoding | AV1 | AV1-RM | 32.063 | 562,84 | 0,0451976 |
| **SPOCK1** | ProteinCoding | AV1 | AV1-RM | 103.025 | 164.816 | 0,0451976 |
| CPA4 | ProteinCoding | AV1 | AV1-RM | 381.981 | 236.798 | 0,0451976 |

*For novel lncRNA and TUCP identified in more samples or by more programs only one transcript name was kept. In red transcript found significant in all samples comparisons.*

***Supplementary Table 5:*** *Melanoma TCGA samples showing MIRAT expression*

| **TCGA sample ID** | **MIRAT Expr (log2)** |
| --- | --- |
| SKCM-Tumor-TCGA-D3-A2J8 | -4.596 |
| SKCM-Tumor-TCGA-DA-A1HW | -4.899 |
| SKCM-Tumor-TCGA-EE-A2GP | -5.185 |
| SKCM-Tumor-TCGA-EE-A2GC | -5.586 |
| SKCM-Tumor-TCGA-EE-A29G | -5.709 |
| SKCM-Tumor-TCGA-FS-A1ZH | -5.743 |
| SKCM-Tumor-TCGA-EE-A2M5 | -5.829 |
| SKCM-Tumor-TCGA-ER-A3ES | -5.902 |
| SKCM-Tumor-TCGA-FS-A1YY | -6.014 |
| SKCM-Tumor-TCGA-EE-A3AG | -6.171 |
| SKCM-Tumor-TCGA-FS-A1ZU | -6.205 |
| SKCM-Tumor-TCGA-DA-A1IB | -6.647 |
| SKCM-Tumor-TCGA-ER-A19J | -6.800 |
| SKCM-Tumor-TCGA-FS-A1ZS | -6.855 |
| SKCM-Tumor-TCGA-DA-A1I1 | -6.893 |
| SKCM-Tumor-TCGA-EE-A2A5 | -6.909 |
| SKCM-Tumor-TCGA-EE-A2MH | -6.912 |
| SKCM-Tumor-TCGA-DA-A1I5 | -6.920 |
| SKCM-Tumor-TCGA-FW-A3I3 | -6.959 |
| SKCM-Tumor-TCGA-EE-A20H | -7.104 |
| SKCM-Tumor-TCGA-D3-A3CC | -7.138 |
| SKCM-Tumor-TCGA-FS-A1ZK | -7.168 |
| SKCM-Tumor-TCGA-FS-A1ZZ | -7.204 |
| SKCM-Tumor-TCGA-D3-A1QA | -7.224 |
| SKCM-Tumor-TCGA-EE-A3J3 | -7.234 |
| SKCM-Tumor-TCGA-EE-A2A2 | -7.271 |
| SKCM-Tumor-TCGA-EE-A29P | -7.324 |
| SKCM-Tumor-TCGA-EE-A3JB | -7.410 |
| SKCM-Tumor-TCGA-EE-A3JI | -7.781 |
| SKCM-Tumor-TCGA-ER-A19L | -7.806 |
| SKCM-Tumor-TCGA-D3-A1Q4 | -7.812 |
| SKCM-Tumor-TCGA-D3-A2J9 | -7.820 |
| SKCM-Tumor-TCGA-EE-A183 | -8.053 |
| SKCM-Tumor-TCGA-FS-A1ZJ | -8.153 |
| SKCM-Tumor-TCGA-D3-A3MV | -8.328 |
| SKCM-Tumor-TCGA-ER-A197 | -8.347 |
| SKCM-Tumor-TCGA-D3-A2JA | -9.494 |
| SKCM-Tumor-TCGA-FS-A1Z4 | -11.025 |
| SKCM-Tumor-TCGA-EE-A181 | -11.399 |
| SKCM-Tumor-TCGA-D3-A2JH | -11.643 |
| SKCM-Tumor-TCGA-EE-A2GD | -11.963 |
| SKCM-Tumor-TCGA-EE-A2GN | -12.510 |

*MIRAT expression levels were calculated using the online available TANRIC software accordingly to described methods*^1^*.*

***Supplementary Table 6:*** *Clinical information of melanoma patients tested for MIRAT expression*

| **Patient ID** | **mutation** | **Age** | **Gender** | **Treatment at time of biopsy** |
| --- | --- | --- | --- | --- |
| **1** | NA | 56 | F | Treatment naive |
| **2** | BRAF WT | 41 | M | Treatment naive |
| **3** | BRAF WT | 46 | F | Previous immunotherapy |
| **4** | BRAF WT | 63 | M | Previous immunotherapy |
| **5** | BRAF V600K | 77 | F | Immunotherapy |
| **6** | BRAF WT | 71 | M | Immunotherapy |
| **7** | BRAF WT | 69 | F | Immunotherapy |
| **8** | BRAF V600R | 78 | M | BRAF inhibitor |
| **9** | BRAF V600K | 57 | F | Treatment naive |
| **10** | BRAF V600E | 64 | M | BRAF and MEK inhibitors |
| **11** | BRAF/NRAS WT | 40 | F | Previous immunotherapy |
| **12** | BRAF V600E | 59 | F | Treatment naive |
| **13** | BRAF WT | 72 | M | MEK and AKT inhibitors |
| **14** | BRAF K601E | 70 | M | BRAF inhibitor |
| **15** | BRAF V600E | 57 | M | BRAF and MEK inhibitors |
| **16** | BRAF/NRAS WT | 41 | F | MEK and AKT inhibitors |
| **17** | NRAS Q61H | 65 | M | MEK and AKT inhibitors |
| **18** | NRASQ61K | 59 | F | MEK and AKT inhibitors |
| **19** | BRAF V600K | 64 | M | BRAF and MEK inhibitors |

**SUPPLEMENTARY MATERIAL AND METHODS**

RNA sequencing and Bioinformatic pipeline

We obtained about 9.3 x 10^7^ reads per sample. We built an initial reference annotation by integrating RefSeq and Gencode data using Cuffcompare to get rid of the redundant transcripts. To identify previously not annotated novel transcripts we used Cufflinks and Scripture for the *de-novo* transcriptome assembly. We filtered possible lncRNA transcripts using the following criteria: a) only transcripts found in more than one condition or in both programs in one or more conditions as identified by cuffcompare were considered for further analysis b)all transcript exons which overlapped any part of any of the exons annotated in the merged reference file consisting of RefSeq and lncRNAs found in Gencode were excluded from further analysis c) all transcripts with length <200 bp and/or intron size of <10bp were excluded from further analysis. We merged all isoforms using cuffmerge. We included the novel genes into our initial references to arrive to the final reference. To calculate FPKM values and its changes for each given gene (coding and noncoding) we compared different conditions using cuffdiff. Because several of the assembled transcripts might be experimental or biological noise we applied an expression threshold (FPKM>0.2) which was defined by the integration of chromatin state data and RNA-data from the ENCODE project. Transcripts under this value were considered as not expressed. Afterwards. we identified transcripts with unknown coding potential (TUPC) by incorporating two methods: i) by analyzing the Coding Potential Assessment Tool (CPAT) and ii) by applying the HMMM3 algorithm.
